# Lewis-base ligand-reshaped interfacial hydrogen-bond network boosts CO_2_ electrolysis

**DOI:** 10.1093/nsr/nwae218

**Published:** 2024-06-22

**Authors:** Wangxin Ge, Haolan Tao, Lei Dong, Yu Fan, Yanpu Niu, Yihua Zhu, Cheng Lian, Honglai Liu, Hongliang Jiang, Chunzhong Li

**Affiliations:** Key Laboratory for Ultrafine Materials of Ministry of Education, School of Chemical Engineering, East China University of Science and Technology, Shanghai 200237, China; Shanghai Engineering Research Center of Hierarchical Nanomaterials, School of Materials Science and Engineering, East China University of Science and Technology, Shanghai 200237, China; State Key Laboratory of Chemical Engineering, Shanghai Engineering Research Center of Hierarchical Nanomaterials, School of Chemistry and Molecular Engineering, East China University of Science and Technology, Shanghai 200237, China; Key Laboratory for Ultrafine Materials of Ministry of Education, School of Chemical Engineering, East China University of Science and Technology, Shanghai 200237, China; Key Laboratory for Ultrafine Materials of Ministry of Education, School of Chemical Engineering, East China University of Science and Technology, Shanghai 200237, China; State Key Laboratory of Chemical Engineering, Shanghai Engineering Research Center of Hierarchical Nanomaterials, School of Chemistry and Molecular Engineering, East China University of Science and Technology, Shanghai 200237, China; Shanghai Engineering Research Center of Hierarchical Nanomaterials, School of Materials Science and Engineering, East China University of Science and Technology, Shanghai 200237, China; State Key Laboratory of Chemical Engineering, Shanghai Engineering Research Center of Hierarchical Nanomaterials, School of Chemistry and Molecular Engineering, East China University of Science and Technology, Shanghai 200237, China; State Key Laboratory of Chemical Engineering, Shanghai Engineering Research Center of Hierarchical Nanomaterials, School of Chemistry and Molecular Engineering, East China University of Science and Technology, Shanghai 200237, China; Key Laboratory for Ultrafine Materials of Ministry of Education, School of Chemical Engineering, East China University of Science and Technology, Shanghai 200237, China; Key Laboratory for Ultrafine Materials of Ministry of Education, School of Chemical Engineering, East China University of Science and Technology, Shanghai 200237, China; Shanghai Engineering Research Center of Hierarchical Nanomaterials, School of Materials Science and Engineering, East China University of Science and Technology, Shanghai 200237, China; Department of Chemical Engineering, School of Chemistry and Chemical Engineering, Shanghai Jiao Tong University, Shanghai 200240, China

**Keywords:** CO_2_ electrolysis, electrode-electrolyte interface, Lewis acid-base interaction, hydrogen-bonding interaction, AIMD simulation

## Abstract

Both the catalyst and electrolyte strongly impact the performance of CO_2_ electrolysis. Despite substantial progress in catalysts, it remains highly challenging to tailor electrolyte compositions and understand their functions at the catalyst interface. Here, we report that the ethylenediaminetetraacetic acid (EDTA) and its analogs, featuring strong Lewis acid-base interaction with metal cations, are selected as electrolyte additives to reshape the catalyst-electrolyte interface for promoting CO_2_ electrolysis. Mechanistic studies reveal that EDTA molecules are dynamically assembled toward interface regions in response to bias potential due to strong Lewis acid-base interaction of EDTA^4–^-K^+^. As a result, the original hydrogen-bond network among interfacial H_2_O is disrupted, and a hydrogen-bond gap layer at the electrified interface is established. The EDTA-reshaped K^+^ solvation structure promotes the protonation of *CO_2_ to *COOH and suppressing *H_2_O dissociation to *H, thereby boosting the co-electrolysis of CO_2_ and H_2_O toward carbon-based products. In particular, when 5 mM of EDTA is added into the electrolytes, the Faradaic efficiency of CO on the commercial Ag nanoparticle catalyst is increased from 57.0% to 90.0% at an industry-relevant current density of 500 mA cm^−2^. More importantly, the Lewis-base ligand-reshaped interface allows a range of catalysts (Ag, Zn, Pd, Bi, Sn, and Cu) to deliver substantially increased selectivity of carbon-based products in both H-type and flow-type electrolysis cells.

## Teaser

This study reports that ethylenediaminetetraacetic acid and its analogs, featuring strong Lewis acid-base interactions with metal cations, are selected as electrolyte additives to reshape electrode-electrolyte interface for boosting CO2 electrolysis.

## INTRODUCTION

Electrocatalysis, powered by electricity produced from renewable energy sources, is central to enable the conversion of earth-abundant molecules (typically H_2_O, O_2_, N_2_ and CO_2_) into carbon-neutral fuels and chemicals [1–5]. The electrode-electrolyte interface where electrocatalytic reactions occur, buried between solid-catalysts and electrolytes, involves complicated processes of electron transfer and mass diffusion under an applied electric field [6,7]. Understanding the interfacial organization and possible interfacial interactions [8–10], such as those between the electrocatalysts and electrolytes or among electrolyte components, is essential for improving electrochemical performance via the co-optimisation of electrocatalysts and electrolytes.

CO_2_ electrolysis has emerged as a promising approach to upgrade rich carbon resources into value-added chemical feedstocks [11,12]. Currently, most of the reported studies focus on developing catalysts with well-designed structure, morphology and composition in order to enhance their intrinsic activities and/or increase the number of active sites [13,14]. However, the role of electrolyte components is equally crucial but extraordinarily under-researched [15,16]. The employment of electrode modification and electrolyte additives at the electrode-electrolyte interface has been shown to enable improved selectivity toward carbon-based products [17–28]. These studies highlight new opportunities to harness electrolytes to steer activity and selectivity. Even so, many open questions remain regarding the molecule-level picture of interfacial organization and dynamic evolution, intrinsic interfacial interactions, and their functions toward electrocatalytic processes [7].

For CO_2_ electrolysis in aqueous electrolytes, H_2_O molecules acting as proton sources are usually involved in the proton-coupled electron transfer (PCET) process, but inevitably lead to an undesired hydrogen evolution reaction (HER) [29,30]. The interfacial hydrogen-bond (H-bond) network is the highway for proton transfer from bulk to electrode surface and therefore greatly influences hydrogen-related reaction kinetics at the electrified interface [26,31–35]. Several recent studies have highlighted the important role of the H-bond network on reaction kinetics for various electrochemical processes. For instance, Li *et al.* revealed the evolution of interfacial H-bonds with changing bias potential, and found that an ordered interfacial water achieved high-efficiency electron transfer for HER [34]. Chen and co-workers demonstrated that the discontinuity of the H-bond network in the alkaline electric double layers (EDLs) resulted in a slow kinetics of hydrogen electrocatalytic reaction [32]. Jia *et al.* reported that the introduction of N-methylimidazoles into electrolytes can restore the interfacial H-bond network to improve hydrogen reaction rates [33]. Nevertheless, the complicated interfacial environment of the co-electrolysis of CO_2_ and H_2_O, at the molecular level, makes understanding the role of the interfacial H-bond network and developing corresponding modulation strategies a great challenge.

In this work, ethylenediaminetetraacetic acid (EDTA) and its analogs were introduced into an aqueous KHCO_3_ electrolyte to reshape the electrified interface, enabling a prominent enhancement of CO_2_ electrolysis to carbon-based products. Nuclear magnetic resonance (NMR) spectroscopy analysis and molecular dynamic (MD) simulations demonstrated the interactions among electrolyte compositions. *In situ* surface-enhanced infrared and *ab initio* molecular dynamics (AIMD) simulations were performed to elucidate the nature of interfacial environment. It was revealed that, when the EDTA molecules were assembled toward the EDL region in response to bias potential, it was able to reconstruct the original interfacial H-bond network via the Lewis acid-base interaction of EDTA^4–^-K^+^. A H-bond gap layer in the EDL region was established, significantly affecting proton transfer kinetics. AIMD simulation provided the information regarding the energy barrier of hydrogen-related reaction kinetics. The EDTA molecules acted on the cationic hydration shell to promote the protonation of *CO_2_ to *COOH and inhibit the rate-limiting Volmer step of HER. We founded that the EDTA-containing electrolyte afforded a kinetically favourable local environment for CO_2_ conversion. Consequently, CO Faradaic efficiency of 90% at 500 mA cm^−2^ was delivered on a commercial Ag catalyst in a flow-type cell. This strategy was universal for improving the carbon-based product selectivity of other catalysts, such as palladium (Pd), zinc (Zn), bismuth (Bi), tin (Sn) and copper (Cu).

## RESULTS AND DISCUSSION

### EDTA-reshaped electrolyte structure

**Figure 1.**
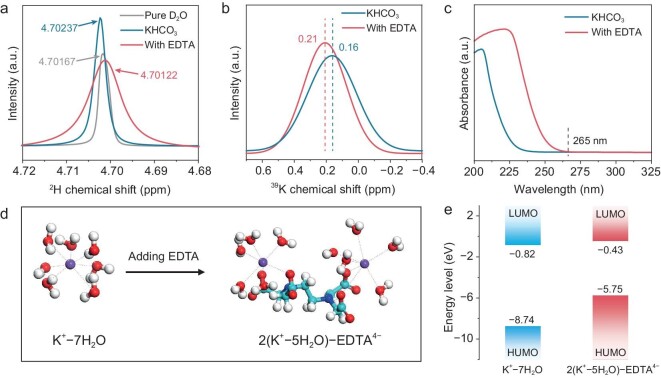
Investigation into electrolyte structure. (a) ^2^H-NMR spectra of the pure D_2_O, KHCO_3_ electrolytes without and with 5 mM EDTA. The electrolyte is prepared by using D_2_O as solvent. (b) ^39^K-NMR spectra of KHCO_3_ electrolytes without and with 5 mM EDTA. (c) UV–vis spectrum of KHCO_3_ electrolytes without and with 5 mM EDTA. (d) Optimized solvation structure of the electrolyte before and after the introduction of EDTA by MD simulation. The O, H, C, N and K are colored in red, white, cyan, blue and purple, respectively. (e) The LUMO and HOMO energies of K^+^-7H_2_O and 2(K^+^-5H_2_O)-EDTA^4−^.

EDTA, a typical Lewis-base ligand, is preferentially selected as an electrolyte additive together with Lewis acid cations to build a composite solvation structure [36]. First, ^13^C NMR spectroscopy was used to probe the structural form of EDTA in the electrolyte. No carboxylate (−COOH) signal is observed in the ^13^C NMR spectrum of the KHCO_3_-EDTA electrolyte (Fig. S1), suggesting that EDTA molecules are completely deprotonated and exist as EDTA^4–^ ions [37]. The deprotonated carboxyl group of EDTA^4–^ is expected to form a strong Lewis acid-base interaction with K^+^, and H-bond interaction with H_2_O. Deuterium (^2^H, D) NMR spectroscopy was performed to investigate EDTA-water interaction in the electrolyte. The ^2^H peak presents a high-field shift and broadening phenomena after the introduction of EDTA to KHCO_3_ electrolyte (Fig. 1a and Fig. S2a), suggesting that the electron density of water is increased by EDTA molecules [38]. An upfield chemical shift of water indicates disruption of the original H-bond network among water molecules by EDTA-water H-bond interaction [39,40]. To further confirm the H-bond interaction of EDTA-water, solutions without KHCO_3_ were also prepared and tested (Fig. S2b), in which the same trend was also observed. The Raman shift signal variations of the O–H stretching vibration (Fig. S3) also evidence this inference.

To validate the Lewis acid-base interaction of EDTA^4–^-K^+^ in the electrolyte, the electrolyte was studied using ^39^K NMR spectroscopy. After the introduction of EDTA, a narrow down-field shift of 0.21 ppm is observed for the electrolyte with EDTA (Fig. 1b), indicating a strong de-shielding effect on the K nucleus [38]. This decreases the content of cation-coordinated water. In other words, the K^+^ solvation structure is changed due to the addition of EDTA. UV-vis spectrum of the electrolyte with EDTA displays an enhanced absorption peak starting from ∼265 nm (Fig. 1c), implying the presence of EDTA^4–^-K^+^ complexes [41,42]. Furthermore, MD simulation and quantum chemical calculation were applied to understand the coordination environment of K^+^ ions in the electrolytes. We performed the MD simulation using the Large-scale Atomic/Molecular Massively Parallel Simulator software package (LAMMPS) to dissect the K^+^ solvation structure in the electrolytes (Fig. S4a). Radial distribution of K–O (H_2_O), K–O (EDTA) and K–N (EDTA) pairs were calculated (Fig. S5). The sharp peaks of the K–O (EDTA) and K–N (EDTA) pairs are identified at 3 Å in the KCl-EDTA electrolyte, which is the same as that of K–O (H_2_O) in the KCl system from the radial distribution function (RDF) analysis, indicating that EDTA participates in the K^+^ solvation structure. The optimal structures of K^+^ ions with and without EDTA were presented (Fig. 1d and Fig. S4b, c), which were used to analyze the electrostatic potential (ESP) mapping and frontier molecular orbital energy using the Gaussian 09 program. The EDTA-reshaped K^+^ solvation structure presents a decreased value of ESP, which suggests a decreased electrostatic repulsion around K^+^, further highlighting the strong Lewis acid-base interaction of EDTA^4–^-K^+^ (Fig. S6) [43]. Moreover, the highest occupied molecular orbital (HOMO) of the 2(K^+^-5H_2_O)-EDTA^4−^ complex displays a higher position than that of the K^+^-7H_2_O (Fig. 1e), indicating that electrons tend to transfer from EDTA^4–^ to K^+^ due to the strong Lewis acid-base interaction between EDTA^4–^ and K^+^ ions [44]. The lowest unoccupied molecular orbital (LUMO) of K^+^-7H_2_O is lower than that of the 2(K^+^-5H_2_O)-EDTA^4−^ complex, indicating lower stability of K^+^-7H_2_O in the electrolyte [44]. The above results indicate that EDTA molecules reconstruct the original H-bond network among H_2_O by EDTA-H_2_O H-bond interaction, and also change the coordination environment of K^+^ ions in electrolytes.

### Effect of EDTA additive on electrocatalytic performance

**Figure 2.**
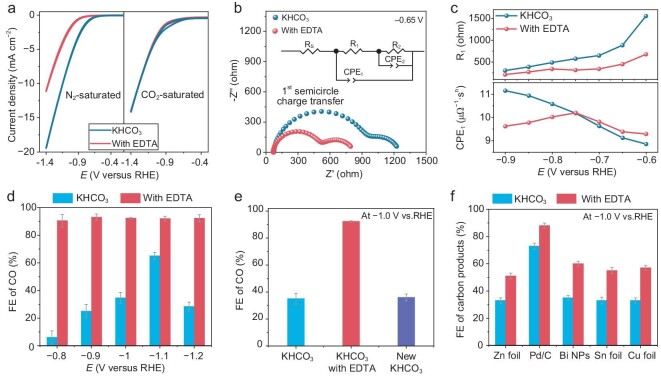
CO_2_ electrolysis performance in KHCO_3_ and KHCO_3_-EDTA electrolytes. (a) CV tests in KHCO_3_ electrolytes with and without 5 mM EDTA at a scan rate of 10 mV s^−1^ under N_2_- and CO_2_-saturated conditions. (b) Nyquist plots of two systems measured at −0.65 V_RHE_. The inset equivalent circuit was used for EIS spectra fitting. (c) Numerical data fitting results of EIS spectra measured at different potentials: R_1_ and CPE_1_. The concentration of KHCO_3_ in the EIS test was 0.5 M, and the added EDTA concentration was 5 mM. (d) FE of CO tested with Ag foil as the electrode in 0.5 M KHCO_3_ electrolytes with and without 5 mM EDTA. (e) FE of CO tested with Ag foil as the electrode in 0.5 M KHCO_3_ electrolytes with and without 5 mM EDTA, and new KHCO_3_ electrolyte at −1.0 V_RHE_. (f) FE of carbon-based products with Zn foil, Pd/C, Bi NPs, Sn foil and Cu foil as the electrode in 0.5 M KHCO_3_ electrolytes with and without 1 mM EDTA at −1.0 V_RHE_. The error bars represent three independent tests.

In order to investigate the effect of Lewis-base EDTA molecules on electrocatalytic reactions, we conducted cyclic voltammograms (CVs) on rotating disk electrodes (RDEs) for Ag electrodes in N_2_- and CO_2_-saturated KHCO_3_ (0.1 M) electrolytes with and without EDTA. As shown in Fig. 2a, in N_2_-saturated electrolytes, the EDTA-containing system shows decreased current density compared with pure KHCO_3_ electrolytes, demonstrating the decreased activity of H_2_O dissociation and the suppression effect of HER in the presence of EDTA. Combined with the above NMR spectroscopy, the drop in HER performance may be attributed to the H-bond formation between EDTA and H_2_O, which leads to a limited proton source for HER [26]. In CO_2_-saturated electrolytes, the current density is higher than that in N_2_-saturated electrolytes with EDTA (Fig. 2a and Fig. S7), implying that the interfacial CO_2_ conversion is indeed promoted after the introduction of EDTA [45,46].

Furthermore, we used electrochemical impedance spectroscopy (EIS) as an interface analysis technique that is capable of probing potential-dependent reaction kinetics, from which charge transfer resistance (R_ct_) and constant-phase elements (CPE) can be extracted and linked to charge transfer kinetics and interfacial ion organization [47,48]. We performed EIS on the RDE electrode near the onset potential (from −0.6 to −0.9 V_RHE_, reversible hydrogen electrode, RHE) to ensure that tests were kinetically controlled, and to avoid as far as possible the effects of bubble disturbance (Fig. S8). A modified Randle's circuit model that includes two possible electrochemical interfaces with different timescales was used (Fig. 2b) [49]. High-frequency intervals (1st semicircle) reflect the kinetic characteristics of electrochemical reactions [50]. Compared with two systems in the high-frequency region, we observe that the EDTA-containing system exhibits a lower R_ct_ (that is R_1_, Fig. 2c), indicating that the electron transfer process is more facile during CO_2_ electroreduction.

The capacitive terms (CPE values) relate to the accumulated ions at or near the electrode-electrolyte interface [48,51]. From –0.6 to –0.75 V_RHE_, the EDTA system exhibits higher CPE_1_ values (Fig. 2c and Tables S1, S2), which is attributed to the assembly of EDTA^4–^ ions bonded with K^+^ ions toward the interface region in response to bias potential, evidencing the existence of Lewis acid-base interaction of EDTA^4–^-K^+^. Particularly, a decrease in CPE_1_ values is observed after the bias potential of –0.75 V_RHE_, which is attributed to the loose alignment that occurs in the outer Helmholtz plane (OHP, a closely packed layer of cations) [52]. The loose alignment of K^+^ ions is caused by repulsive interaction of the electric field toward EDTA^4–^ ions under a higher reduction potential, further suggesting the strong interaction of EDTA^4–^-K^+^.

Electrocatalytic performance was measured at −0.8 to −1.2 V_RHE_ in CO_2_-saturated 0.5 M KHCO_3_ electrolytes with and without EDTA in H-type cells (Fig. 2d and Figs S9, S10). In the electrolyte with 1 mM EDTA, a commercial Ag foil electrode delivered a much higher CO Faradaic efficiency (FE) and partial current density (92% and 11.5 mA cm^−2^) than those (34% and 3 mA cm^−2^) in the EDTA-free electrolyte at −1.0 V_RHE_, highlighting the key role of EDTA additives on boosting the selectivity of CO_2_ reduction to CO. The Staircase voltammetry (SCV) tests of both electrolyte systems also clearly illustrate the inhibition of HER and acceleration of CO_2_RR by EDTA (Fig. S11). The commercial Ag nanoparticle (NP)-supported carbon paper electrode shows similar performance trends (Fig. S12). The Ag foil electrode in the EDTA-containing electrolyte also exhibits decent stability for CO_2_ electroreduction to CO (Fig. S13). In addition, we fixed the same Ag electrode and tested it successively in different electrolytes. After replacing the new KHCO_3_ electrolyte, the Ag electrode recovers its original performance (Fig. 2e). This suggests that the elevated catalytic activity is not due to irreversible adsorption [19–23], but may originate from the change of interfacial microenvironment through a non-adsorbed form of EDTA.

To verify the universality of EDTA additives on CO_2_ electrolysis performance, other metal catalysts are also studied. Pd and Zn have been demonstrated to possess decent activities for CO_2_ conversion to CO. Both the metal catalysts display obvious FE_CO_ enhancement (Fig. 2f and Figs S14, S15), wherein a Pd/C catalyst achieves 88% FE_CO_ at −1 V_RHE_ in the EDTA-containing electrolyte. We also explored whether EDTA has the same effect on catalysts toward formate productuction. Both Bi and Sn, common catalysts for the preparation of formate, show significant FE_formate_ enhancement in the EDTA-containing electrolyte (Fig. 2f and Figs S16, S17), whereby the Sn foil catalyst achieves 80% FE_formate_ at −1.0 V_RHE_. Furthermore, we followed on to explore its potential for multi-carbon products (C_2+_) formation on Cu catalysts (Figs S18 and S19). The commercial Cu foil shows a decreased FE of H_2_, and the FE of carbon-based products increased from 33% to 57% at −1.0 V_RHE_ (Fig. 2f and Fig. S18).

To exclude the change of catalyst structure that may impact performance [53,54], a series of characterizations were carried out, showing that the EDTA in the electrolyte does not induce the change of morphology, crystallinity, and oxidation states of the Ag electrode (Figs S20–S22). We also evaluated the stability of EDTA under operational conditions by NMR analysis. The chemical structure of EDTA does not change after electrolysis (Fig. S23).

### *In-situ* probing of the electrified interface

**Figure 3.**
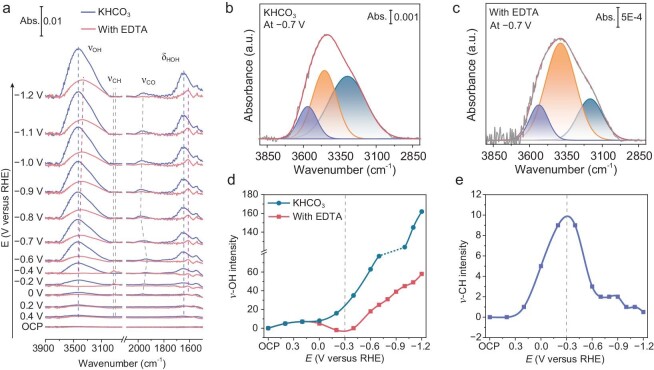
*In-situ* probing electrified interface. (a) *In situ* ATR-SEIRAS spectra under various potentials for the KHCO_3_ electrolyte with and without 1 mM EDTA. (b) Deconvolution of the *ν*-OH peak in 0.5 M KHCO_3_ electrolyte at −0.7 V_RHE_. (c) Deconvolution of the *ν*-OH peak in 0.5 M KHCO_3_ electrolyte with 1 mM EDTA at −0.7 V_RHE_. (d) Intensity of *ν*-OH peaks of KHCO_3_ and EDTA-containing electrolytes under various potentials. (e) Intensity of *ν*-CH peaks of EDTA-containing electrolytes under various potentials.

To gain insights into the electrified interfacial H-bond network, *in situ* attenuated total reflection surface-enhanced infrared absorption spectroscopy (ATR-SEIRAS) was applied (Fig. S24). Figure 3a and Fig. S24 show *in situ* ATR-SEIRAS spectra on Ag at various potentials in CO_2_-saturated 0.5 M KHCO_3_ with and without 1 mM EDTA, respectively. For two electrolyte systems, several vibrational bands were found to appear at 3650—3000, 2950—2800, 2000—1850 and 1650—1610 cm**^−^**^1^, which can be assigned to O–H stretching mode (ν-OH) of H_2_O [55,56], C–H stretching mode (ν-CH) of EDTA, C–O stretching mode (ν-CO) of CO adsorbed on the top or bridge sites of Ag surface [57,58], and H–O–H bending mode (δ-HOH) of H_2_O, respectively.

First, we analyzed the H-bond environment of the interfacial water. After the introduction of EDTA, the vibration of δ-HOH shifts from 1647 to 1618 cm^−1^ (Fig. 3a), indicating the formation of a weak H-bond network among the interfacial water molecules [56]. The red shift of ν-OH can be attributed to the presence of a stronger H-bond interaction among water molecules or from other electrolyte components [59]. Considering the weak H-bonds among interfacial water, we attribute the red shift of ν-OH in the EDTA-containing system to the strong H-bonds between EDTA and H_2_O. In addition, the ν-OH peak in two electrolyte systems can be deconvoluted into three distinct components through Gaussian fitting [54,60] (Fig. 3b, c and Figs S26, S27), corresponding to a strong H-bond at ∼3250 cm^−1^, medium H-bond at ∼3450 cm^−1^, and weak H-bond at ∼3600 cm^−1^, respectively. Specifically, the ν-OH is dominated by a strong H-bond in the KHCO_3_ system (Fig. 3b). But the medium H-bond is dominant after introducing EDTA (Fig. 3c). This is attributed to the spatial effect of EDTA molecules and the H-bond effect on water disrupting the initial interfacial H-bond network among water. Therefore, the original H-bond network among interfacial H_2_O is reconstructed by EDTA, forming a weakened H-bond network at the electrified interface. The weakened H-bond network can result in an increased proton transfer barrier in the interfacial region [32].

To further explore the interfacial interaction among electrolyte components, we used the absorbance intensities of ν-OH and ν-CH to identify the interfacial content of H_2_O and EDTA in response to bias potential, respectively (Fig. 3d and e). For the pure KHCO_3_ system, the intensity of ν-OH enhanced with increasing bias potential, suggesting that water molecules are gradually adsorbed on the electrode surface from open circuit potential (OCP) to −1.2 V_RHE_. In contrast, for the EDTA system, the intensity of ν-OH appears to first decrease and then increase with increasing bias potential, exhibiting a minimum value at −0.3 V_RHE_. Notably, from OCP to −0.3 V_RHE_, a gradual increase of EDTA at the interface is accompanied by a decrease in the adsorbed water content. Along with the electrostatic effects of K^+^ cations with the electrode, EDTA molecules are adsorbed together by strong Lewis acid-base interactions. At the same time, water molecules are difficult to be quickly replenished to the catalyst surface due to the spatial effect of EDTA. From −0.3 to −1.2 V_RHE_, the amount of EDTA adsorbed at the interface gradually decreases, which is attributed to increased electrostatic repulsion between the electrode surface and the EDTA^4–^ ions. However, the amount of water increases with increasing bias potential, which is attributed to enhanced interfacial electrostatic adsorption between water and electrode [58]. The Raman shift signal variations of the C–H stretching vibration (Fig. S28) also confirm this feature of EDTA distribution under external electric field. From these *in situ* spectra results in conjunction with above EIS analysis, it is revealed that EDTA molecules are assembled toward the interface region in response to bias potential, leading to weakening of the interfacial H-bond network.

### Theoretical investigation into EDTA-reshaped interface

**Figure 4.**
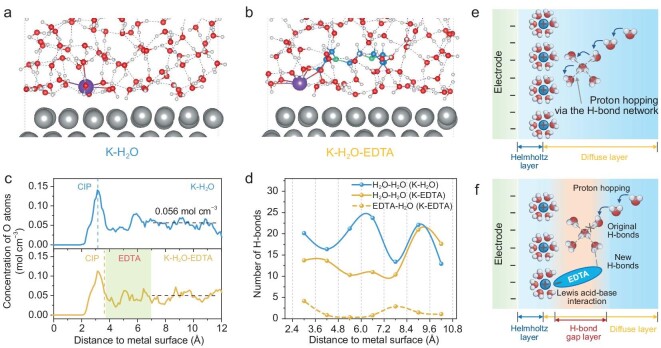
EDL structures of K-H_2_O and K-H_2_O-EDTA interfaces. Representative snapshots of EDL structures on the Ag(111) electrode surface for K-H_2_O system (a) and K-H_2_O-EDTA system (b). The Ag, O, H, C, N and K are colored in grey, red, white, blue, green and purple, respectively. The blue dashed lines represent the H-bonds. (c) Concentration distribution profiles of O atoms in water and EDTA along the surface normal direction. The vertical dashed lines represent the CIPs. The area marked in green is the location of the EDTA molecule. The horizontal black dashed lines represent the bulk water concentration (0.056 mol cm^–3^). (d) Statistical distribution of H-bond number along the surface normal direction for K-H_2_O and K-H_2_O-EDTA systems. (e) Schematic of interfacial H-bond network for EDTA-free electrolyte. (f) Schematic of interfacial H-bond network and the proposed regulation mechanism for EDTA-containing electrolyte.

To further provide in-depth insights into the EDTA-reconstructed interface, we conducted AIMD simulation to study the EDL structures of K-H_2_O and K-H_2_O-EDTA interfaces on an Ag(111) supercell (Fig. S29). Figure 4a and b shows the representative AIMD snapshots for the EDL structures of K-H_2_O and K-H_2_O-EDTA configurations, respectively. The planes consisting of the cations closest to the electrode surface are defined as the closest ion planes (CIPs), which are at distances of ∼3.27 Å and ∼3.72 Å away from the electrode surface for K-H_2_O and K-H_2_O-EDTA systems, respectively. The cations at the K-H_2_O-EDTA system lose some solvated molecules (Fig. 4b), which is due to the participation of EDTA molecules in K^+^ solvation structure. The CIP shift away from surface normal direction is attributed to the Lewis acid-base interaction between EDTA^4–^ and K^+^. The cation and water distributions in the two systems suggest that the CIPs correspond to the outer Helmholtz plane at the interface, which agrees with previous work about the alkaline interface [32]. The interfacial oxygen concentration profiles along the surface normal direction were studied for the distribution of water molecules and interaction of EDTA-H_2_O (Fig. 4c). Since oxygen atoms are mainly contributed by water molecules, the significantly lower oxygen concentration in the EDTA system indicates that the introduction of EDTA brings about a low content of interfacial water, which matches the results of the above *in situ* experiments. The decrease in the concentration of water molecules would reduce connectivity of the H-bond network in the EDL region, which is verified by the statistical distribution of H-bonds along the surface normal direction (Fig. 4d). The statistics for H-bond number in the K-H_2_O system are similar to previous reports [32]. In the K-H_2_O-EDTA system, the H-bond number is the sum of the H_2_O-H_2_O H-bonds and the EDTA-H_2_O H-bonds. Over a large spatial range from 3.0 to 7.8 Å, the H_2_O-H_2_O H-bond number is significantly lower than that of the K-H_2_O system, forming a H-bond gap layer in the EDL region (Fig. 4e and f). The H-bond gap layer decreases the connectivity of the H-bond network and can affect hydrogen-related reaction kinetics [32].

**Figure 5.**
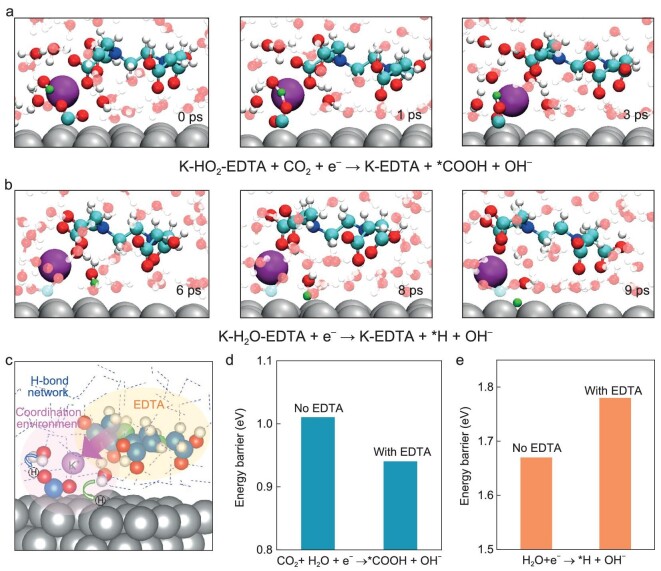
Theoretical insights into hydrogen-related electrocatalytic processes at K-H_2_O and K-H_2_O-EDTA interfaces. (a) Representative snapshots of the *CO_2_ to *COOH step at K-H_2_O-EDTA interface. (b) Representative snapshots of the Volmer step at K-H_2_O-EDTA interface. The Ag, O, H, C, N and K are colored in grey, red, white, cyan, blue and purple, respectively. The green hydrogen atoms represent those involved in hydrogen-related electrocatalytic processes. (c) Schematic illustration of the EDTA effect on the cationic coordination environment, where in the H-bond network the processes of CO_2_ protonation and H_2_O dissociation occurs. The Ag, O, H, C, N and K are colored in grey, red, white, sky blue, green and purple, respectively. (d) Energy barrier of the *CO_2_ to *COOH step at K-H_2_O and K-H_2_O-EDTA interfaces. (e) Energy barrier of the Volmer step at K-H_2_O and K-H_2_O-EDTA interfaces.

The EDTA has a direct impact on the first-solvated shell of cations at CIPs, and may alter the reaction kinetics of hydrogen-related electrocatalytic processes. After introducing a CO_2_ molecule into the K-H_2_O and K-H_2_O-EDTA systems (Figs S30 and S31), we further performed constrained AIMD (cAIMD) simulations to investigate the effect of EDTA on the free energy of the proton transfer processes. The cAIMD method was performed to evaluate the cation-coordinated CO_2_ electroreduction on Ag-water interfaces [61–63]. Based on the obtained interface pictures (Fig. 4a and b), the CO_2_ and H_2_O molecules in the cationic coordination environment were selected for cAIMD calculations in the two systems with and without EDTA. We first simulated the *CO_2_ to *COOH conversion process in both EDTA and EDTA-free systems, where the water was the proton donor (Fig. 5a and Fig. S32a). Afterward, for the rate-determining Volmer step of HER (*H_2_O to *H), both EDTA and EDTA-free systems were also considered (Fig. 5b and Fig. S32b). According to these snapshots of species evolution, it is found that the EDTA undergoes a cation-mediated pathway to affect the proton transfer steps, when the interplay between the EDTA and the cationic coordination environment occurs in the H-bond network (Fig. 5c). For the K-H_2_O-EDTA-CO_2_ system, the free energy of *CO_2_ to *COOH step (0.94 eV) is lower than the EDTA-free system (1.01 eV), indicating that the EDTA promotes this cation-mediated proton transfer process (Fig. 5d and Fig. S33a, b). With K-H_2_O-EDTA system, the kinetic barrier of *H_2_O to *H is 1.78 eV, higher than the 1.67 eV of the EDTA-free system (Fig. 5e and Fig. S33c, d), demonstrating that EDTA-containing cation shell leads to poor HER activity via suppressing water dissociation. Eventually, by regulating the proton transfer kinetics of cation-coordinated water, in EDTA-containing systems, CO_2_ reduction is facile with the lower energy barrier for the protonation of *CO_2_ to *COOH. Meanwhile, HER is suppressed, showing a higher energy barrier in the rate-limiting Volmer step.

### Lewis-base ligands enabling the boosting CO_2_ electrolysis in flow-type cells

**Figure 6.**
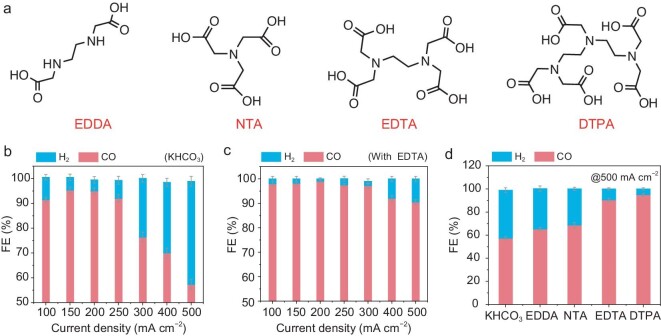
CO_2_ electrolysis performance in a flow-type cell. (a) Structural formulae of EDDA, NTA, EDTA and DTPA. (b) Faradaic efficiency of H_2_ and CO at various current densities in 1 M KHCO_3_ electrolytes. (c) Faradaic efficiency of H_2_ and CO at various current densities in 1 M KHCO_3_ electrolytes with 5 mM EDTA. (d) Faradaic efficiency of H_2_ and CO at 500 mA cm^–2^ in 1 M KHCO_3_ electrolytes without and with different Lewis base molecules. The error bars represent three independent tests.

To evaluate the benefits of EDTA additives for high-rate CO_2_ electrolysis at commercially relevant performance metrics, we introduced the EDTA to the electrolytes in flow-type cells equipped with gas diffusion electrodes (GDE) (Figs S34, S35). Over full current density range (100−500 mA cm^−2^), the EDTA-containing system greatly improves FE_CO_ (Fig. 6) and also delivers good stability (Fig. S36). These results suggest that a high FE_CO_ is still achieved in flow cells with the EDTA additive. The commercial Cu NPs were also tested in flow-type cells, and similar results of the boosted FE of carbon-based products were shown (Fig. S37).

In response to bias potential, EDTA molecules are assembled toward the EDL region via the Lewis acid-base interaction between deprotonated carboxyl groups and K^+^ ions [36]. This leads to the formation of a favourable interface environment for CO_2_ conversion. To further expand this concept, we also employed EDTA analogs as electrolyte additives, including EDDA, NTA and DTPA (where EDDA is ethylenediamine-*N, N*′-diacetic acid, NTA is nitrilotriacetic acid, DTPA is diethylenetriaminepentaacetic acid). The number of carboxyl groups for EDDA, NTA, EDTA and DTPA is 2, 3, 4 and 5, respectively (Fig. 6a). All these ligand systems compared to the pure KHCO_3_ system show an increased FE_CO_ in flow-type cells with the cathode area of 1 and 10 cm^2^ (Fig. 6d and Figs S38–S41). The FE_CO_ increases with increasing number of carboxyl groups. This is because more carboxyl groups result in a more significant disruption of the H-bond network among H_2_O molecules, as evidenced by ^1^H-NMR spectra of the different additive molecules (Fig. S42). Notably, the shift degree of the water peak (Fig. S41) toward high field is consistent with the trend of FE_CO_ (Fig. 6d), highlighting that modulation of the H-bond network is closely associated with the delivered FE_CO_. The DTPA system compared to the EDTA exhibits slightly higher FE_CO_ (Fig. 6d). It should be pointed out that the DTPA compared to the EDTA exhibits lower water solubility and higher cost, implying that the EDTA may be optimal among these Lewis-base ligands.

## CONCLUSION

In summary, by virtue of comprehensive *in situ* vibrational spectroscopic characterizations and AIMD simulations, we have provided molecule-level insights into the Lewis-base ligand-reshaped interfacial organization for promoting CO_2_ electrolysis. EDTA, a typical Lewis-base ligand, has been introduced to the electrolyte to construct a H-bond gap layer in the EDL region, resulting in a lower proton source and a decreased activity of undesired HER. The EDTA enters the K^+^ solvation structure via the strong Lewis acid-base interaction of EDTA^4–^-K^+^. This facilitates the protonation step of *CO_2_ to *COOH while suppressing *H_2_O dissociation to *H. The reconstructed interface in flow cells has enabled commercial Ag nanoparticle catalyst to deliver over 99% and 90% FE_CO_ for CO_2_ electrolysis at −200 and −500 mA cm^−2^, respectively. This electrolyte regulation strategy is universally applicable to different metal catalysts (such as Ag, Zn, Pd, Bi, Sn and Cu) and other similar Lewis-base ligands (such as EDDA, NTA and DTPA). The findings established here propose future directions that electrocatalytic performance of various electrochemical reactions can effectively be steered by tailoring the interactions among ions and/or molecules in electrolytes.

## Supplementary Material

nwae218_Supplemental_File

## References

[1] Cheng T, Yao Z, Mietek J et al. Electrocatalytic refinery for sustainable production of fuels and chemicals. Angew Chem Int Ed 2021; 60: 19572. 10.1002/anie.202101522 33606339

[2] Seh ZW, Kibsgaard J, Dickens CF et al. Combining theory and experiment in electrocatalysis: insights into materials design. Science 2017; 355: 4998. 10.1126/science.aad4998 28082532

[3] Jin S, Hao Z, Zhang K et al. Advances and challenges for the electrochemical reduction of CO_2_ to CO: from fundamentals to industrialization. Angew Chem Int Ed 2021; 60: 20627–48. 10.1002/anie.202101818 33861487

[4] Wang G, Chen J, Ding Y et al. Electrocatalysis for CO_2_ conversion: from fundamentals to value-added products. Chem Soc Rev 2021; 50: 4993–5061. 10.1039/D0CS00071J 33625419

[5] Huang Z, Zhu L, Li A et al. Renewable synthetic fuel: turning carbon dioxide back into fuel. Front Energy 2022; 16: 145–9. 10.1007/s11708-022-0828-6

[6] Sa YJ, Lee CW, Lee SY et al. Catalyst-electrolyte interface chemistry for electrochemical CO_2_ reduction. Chem Soc Rev 2020; 49: 6632–65. 10.1039/D0CS00030B 32780048

[7] Wang JL, Tan HY, Qi MY et al. Spatially and temporally understanding dynamic solid–electrolyte interfaces in carbon dioxide electroreduction. Chem Soc Rev 2023; 52: 5013–50. 10.1039/D2CS00441K 37431250

[8] Wagner A, Sahm CD, Reisner E. Towards molecular understanding of local chemical environment effects in electro- and photocatalytic CO_2_ reduction. Nat Catal 2020; 3: 775–86. 10.1038/s41929-020-00512-x

[9] Wen G, Ren B, Liu Y et al. Bridging trans-scale electrode engineering for mass CO_2_ electrolysis. JACS Au 2023; 3: 2046–61. 10.1021/jacsau.3c00174 37654582 PMC10466330

[10] Yu J, Yin J, Li R et al. Interfacial electric field effect on electrochemical carbon dioxide reduction reaction. Chem Catal 2022; 2: 2229–52. 10.1016/j.checat.2022.07.024

[11] Nitopi S, Bertheussen E, Scott SB et al. Progress and perspectives of electrochemical CO_2_ reduction on copper in aqueous electrolyte. Chem Rev 2019; 119: 7610–72. 10.1021/acs.chemrev.8b00705 31117420

[12] Ma W, He X, Wang W et al. Electrocatalytic reduction of CO_2_ and CO to multi-carbon compounds over Cu-based catalysts. Chem Soc Rev 2021; 50: 12897–914. 10.1039/D1CS00535A 34609390

[13] Wang Y, Han P, Lv X et al. Defect and interface engineering for aqueous electrocatalytic CO_2_ reduction. Joule 2018; 2: 2551–82. 10.1016/j.joule.2018.09.021

[14] Wu Z-Z, Gao F-Y, Gao M-R. Regulating the oxidation state of nanomaterials for electrocatalytic CO_2_ reduction. Energy Environ Sci 2021; 14: 1121–39. 10.1039/D0EE02747B

[15] Xu A, Govindarajan N, Kastlunger G et al. Theories for electrolyte effects in CO_2_ electroreduction. Acc Chem Res 2022; 55: 495–503. 10.1021/acs.accounts.1c00679 35107967

[16] Deng B, Huang M, Zhao X et al. Interfacial electrolyte effects on electrocatalytic CO_2_ reduction. ACS Catal 2021; 12: 331–62. 10.1021/acscatal.1c03501

[17] Zhu Q, Murphy CJ, Baker LR. Opportunities for electrocatalytic CO_2_ reduction enabled by surface ligands. J Am Chem Soc 2022; 144: 2829–40. 10.1021/jacs.1c11500 35137579

[18] Nam D-H, De Luna P, Rosas-Hernández A et al. Molecular enhancement of heterogeneous CO_2_ reduction. Nat Mater 2020; 19: 266–76. 10.1038/s41563-020-0610-2 32099112

[19] Ge W, Chen Y, Fan Y et al. Dynamically formed surfactant assembly at the electrified electrode-electrolyte interface boosting CO_2_ electroreduction. J Am Chem Soc 2022; 144: 6613–22. 10.1021/jacs.2c02486 35380035

[20] Buckley AK, Cheng T, Oh MH et al. Approaching 100% selectivity at low potential on Ag for electrochemical CO_2_ reduction to CO using a surface additive. ACS Catal 2021; 11: 9034–42. 10.1021/acscatal.1c00830

[21] Zhao Y, Xu J, Huang K et al. Dopant- and surfactant-tuned electrode-electrolyte interface enabling efficient alkynol semi-hydrogenation. J Am Chem Soc 2023; 145: 6516–25. 10.1021/jacs.3c00565 36913524

[22] Yang J, Kang X, Jiao J et al. Ternary ionic-liquid-based electrolyte enables efficient electro-reduction of CO_2_ over bulk metal electrodes. J Am Chem Soc 2023; 145: 11512–7. 10.1021/jacs.3c03259 37196054

[23] Sha Y, Zhang J, Cheng X et al. Anchoring ionic liquid in copper electrocatalyst for improving CO_2_ conversion to ethylene. Angew Chem Int Ed 2022; 134: e202200039. 10.1002/ange.202200039 35076980

[24] Liu B, Guo W, Gebbie MA. Tuning ionic screening to accelerate electrochemical CO_2_ reduction in ionic liquid electrolytes. ACS Catal 2022; 12: 9706–16. 10.1021/acscatal.2c02154

[25] Ni W, Guan Y, Chen H et al. Molecular engineering of cation solvation structure for highly selective carbon dioxide electroreduction. Angew Chem Int Ed 2023; 62: e202303233. 10.1002/anie.202303233 37507348

[26] Mohandas N, Narayanan TN, Cuesta A. Tailoring the interfacial water structure by electrolyte engineering for selective electrocatalytic reduction of carbon dioxide. ACS Catal 2023; 13: 8384–93. 10.1021/acscatal.3c01223

[27] Ji K, Liu Y, Wang Y et al. Steering selectivity in electrocatalytic furfural reduction via electrode-electrolyte interface modification. J Am Chem Soc 2024; 146: 11876–86. 10.1021/jacs.4c00818 38626315

[28] Kong K, Li AZ, Wang Y et al. Electrochemical carbon-carbon coupling with enhanced activity and racemate stereoselectivity by microenvironment regulation. Nat Commun 2023; 14: 6925. 10.1038/s41467-023-42724-2 37903827 PMC10616095

[29] Hui SR, De Luna P. How increasing proton and electron conduction benefits electrocatalytic CO_2_ reduction. Matter 2021; 4: 1555–77. 10.1016/j.matt.2021.02.021

[30] Yang P-P, Gao M-R. Enrichment of reactants and intermediates for electrocatalytic CO_2_ reduction. Chem Soc Rev 2023; 52: 4343–80. 10.1039/D2CS00849A 37318005

[31] Li XY, Wang T, Cai YC et al. Mechanism of cations suppressing proton diffusion kinetics for electrocatalysis. Angew Chem Int Ed 2023; 62: e202218669. 10.1002/anie.202218669 36762956

[32] Li P, Jiang Y, Hu Y et al. Hydrogen bond network connectivity in the electric double layer dominates the kinetic pH effect in hydrogen electrocatalysis on Pt. Nat Catal 2022; 5: 900–11. 10.1038/s41929-022-00846-8

[33] Sun Q, Oliverira NJ, Kwon S et al. Understanding hydrogen electrocatalysis by probing the hydrogen-bond network of water at the electrified Pt–solution interface. Nat Energy 2023; 8: 859–69. 10.1038/s41560-023-01302-y

[34] Wang Y-H, Zheng S, Yang W-M et al. *In situ* Raman spectroscopy reveals the structure and dissociation of interfacial water. Nature 2021; 600: 81–5. 10.1038/s41586-021-04068-z 34853456

[35] Li CY, Le J B, Wang YH et al. *In situ* probing electrified interfacial water structures at atomically flat surfaces. Nat Mater 2019; 18: 697–701. 10.1038/s41563-019-0356-x 31036960

[36] Ou Y, Cai Z, Wang J et al. Reversible aqueous Zn battery anode enabled by a stable complexation adsorbent interface. EcoMat 2022; 4: e12167. 10.1002/eom2.12167

[37] Doherty TAS, Nagane S, Kubicki DJ et al. Stabilized tilted-octahedra halide perovskites inhibit local formation of performance-limiting phases. Science 2021; 374: 1598–605. 10.1126/science.abl4890 34941391

[38] Dubouis N, Serva A, Salager E et al. The fate of water at the electrochemical interfaces: electrochemical behavior of free water versus coordinating water. J Phys Chem Lett 2018; 9: 6683–8. 10.1021/acs.jpclett.8b03066 30398885

[39] Zhou L, Wang F, Yang F et al. Unshared pair electrons of zincophilic Lewis base enable long-life zn anodes under “three high” conditions. Angew Chem Int Ed 2022; 61: e202208051. 10.1002/anie.202208051 35971572

[40] Liu TT, Wu H, Du XF et al. Water-locked eutectic electrolyte enables long-cycling aqueous sodium-ion batteries. ACS Appl Mater Interfaces 2022; 14: 33041–51. 10.1021/acsami.2c04893 35849540

[41] Meng R, Li H, Lu Z et al. Tuning zn-ion solvation chemistry with chelating ligands toward stable aqueous zn anodes. Adv Mater 2022; 34: e2200677. 10.1002/adma.202200677 35901291

[42] Virtapohja J . Fate of chelating agents used in the pulp and paper industries. O Papel (Brazil) 1998; 60: 48–54.

[43] Yang J, Yan H, Hao H et al. Synergetic modulation on solvation structure and electrode interface enables a highly reversible zinc anode for zinc–iron flow batteries. ACS Energy Lett 2022; 7: 2331–9. 10.1021/acsenergylett.2c00560

[44] Yu H, Chen D, Ni X et al. Reversible adsorption with oriented arrangement of a zwitterionic additive stabilizes electrodes for ultralong-life Zn-ion batteries. Energy Environ Sci 2023; 16: 2684–95. 10.1039/D3EE00982C

[45] Bondue CJ, Graf M, Goyal A et al. Suppression of hydrogen evolution in acidic electrolytes by electrochemical CO_2_ reduction. J Am Chem Soc 2020; 143: 279–85. 10.1021/jacs.0c10397 33356205 PMC7809687

[46] Dong L, Ge WX, Fan Y et al. Surfactant-modified electrode-electrolyte interface for steering CO_2_ electrolysis on Cu electrodes. AIChE J 2024; 70: e18271. 10.1002/aic.18271

[47] Lazanas AC, Prodromidis MI. Electrochemical impedance spectroscopy-a tutorial. ACS Meas Sci Au 2023; 3: 162–93. 10.1021/acsmeasuresciau.2c00070 37360038 PMC10288619

[48] Wang S, Zhang J, Gharbi O et al. Electrochemical impedance spectroscopy. Nat Rev Methods Primers 2021; 1: 41. 10.1038/s43586-021-00039-w

[49] Lim CYJ, Yilmaz M, Arce-Ramos JM et al. Surface charge as activity descriptors for electrochemical CO_2_ reduction to multi-carbon products on organic-functionalised Cu. Nat Commun 2023; 14: 335. 10.1038/s41467-023-35912-7 36670095 PMC9860078

[50] Zhao Y, Liu X, Chen J et al. Promote electroreduction of CO_2_ via catalyst valence state manipulation by surface-capping ligand. P Natl Acad Sci USA 2023; 120: e2218040120. 10.1073/pnas.2218040120 PMC10235936 37216512

[51] Pajkossy T . Impedance spectroscopy at interfaces of metals and aqueous solutions—surface roughness, CPE and related issues. Solid State Ionics 2005; 176: 1997–2003. 10.1016/j.ssi.2004.06.023

[52] Bandarenka AS . Exploring the interfaces between metal electrodes and aqueous electrolytes with electrochemical impedance spectroscopy. Analyst 2013; 138: 5540–54. 10.1039/c3an00791j 23888300

[53] Lai W, Qiao Y, Zhang J et al. Design strategies for markedly enhancing energy efficiency in the electrocatalytic CO_2_ reduction reaction. Energy Environ Sci 2022; 15: 3603–29. 10.1039/D2EE00472K

[54] Zhang WJ, Hu Y, Ma LB et al. Progress and perspective of electrocatalytic CO_2_ reduction for renewable carbonaceous fuels and chemicals. Adv Sci 2018; 5: 1700275. 10.1002/advs.201700275 PMC5770696 29375961

[55] Rao RR, Huang B, Katayama Y et al. pH- and cation-dependent water oxidation on rutile RuO_2_(110). J Phys Chem C 2021; 125: 8195–207. 10.1021/acs.jpcc.1c00413

[56] Ataka K-i, Yotsuyanagi T, Osawa M. Potential-dependent reorientation of water molecules at an electrode/electrolyte interface studied by surface-enhanced infrared absorption spectroscopy. J Phys Chem 1996; 100: 10664–72. 10.1021/jp953636z

[57] Firet NJ, Smith WA. Probing the reaction mechanism of CO_2_ electroreduction over Ag films via operando infrared spectroscopy. ACS Catal 2017; 7: 606–12. 10.1021/acscatal.6b02382

[58] Katayama Y, Nattino F, Giordano L et al. An In situ surface-enhanced infrared absorption spectroscopy study of electrochemical CO_2_ reduction: selectivity dependence on surface C-bound and O-bound reaction intermediates. J Phys Chem C 2019; 123: 5951–63. 10.1021/acs.jpcc.8b09598

[59] Nihonyanagi S, Yamaguchi S, Tahara T. Counterion effect on interfacial water at charged interfaces and its relevance to the Hofmeister series. J Am Chem Soc 2014; 136: 6155–8. 10.1021/ja412952y 24742093

[60] Rajapriya Inbaraj N, Song S, Chang R et al. Investigation of hydration states of ionic liquids by fourier transform infrared absorption spectroscopy: relevance to stabilization of protein molecules. Langmuir 2023; 39: 2558–68. 10.1021/acs.langmuir.2c02851 36753569 PMC9948542

[61] Qin X, Hansen HA, Honkala K et al. Cation-induced changes in the inner- and outer-sphere mechanisms of electrocatalytic CO_2_ reduction. Nat Commun 2023; 14: 7607. 10.1038/s41467-023-43300-4 37993426 PMC10665450

[62] Qin X, Vegge T, Hansen HA. Cation-coordinated inner-sphere CO_2_ electroreduction at Au-water interfaces. J Am Chem Soc 2023; 145: 1897–905. 10.1021/jacs.2c11643 36630567

[63] Qin X, Vegge T, Hansen HA. CO_2_ activation at Au(110)–water interfaces: an ab initio molecular dynamics study. J Chem Phys 2021; 155: 134703. 10.1063/5.0066196 34624986

