# Supplementary material for: Lewis-base ligand-reshaped interfacial hydrogen-bond network boosts CO2 electrolysis
Source: Natl Sci Rev. 2024 Jun 22;11(8):nwae218. doi: 10.1093/nsr/nwae218 (PMC11259048; doi:10.1093/nsr/nwae218)
Supplement: nwae218_Supplemental_File [file nwae218_supplemental_file.docx]

Supporting Information for

**Lewis-Base Ligand-Reshaped Interfacial Hydrogen-Bond Network Boosts CO_2_ Electrolysis**

Wangxin Ge, Haolan Tao, Lei Dong, Yu Fan, Yanpu Niu, Yihua Zhu, Cheng Lian, Honglai Liu, Hongliang Jiang, and Chunzhong Li

Correspondence to: liancheng@ecust.edu.cn (Cheng Lian); jhlworld@ecust.edu.cn (Hongliang Jiang); czli@ecust.edu.cn and czlilab@sjtu.edu.cn (Chunzhong Li)

**This PDF file includes:**

Experimental procedures

Computational methods

Figures S1 to S42

Tables S1 to S2

1. **Experimental procedures**

1.1 Chemicals

KHCO_3_ (Analytical Reagent (AR), 99.99%), EDTA (AR, 99.99%), DTPA (AR, 99.99%) EDDA (AR, 99.99%), NTA (AR, 99.99%), and Nafion perfluorinated resin solution (5 wt% in a mixture of lower aliphatic alcohols and water) were purchased from Sigma-Aldrich. All chemicals were used as received without further purification. Deionized water (18.25 MΩ cm^−1^) was used throughout all the experiments.

1.2 Electrolyte characterization

Liquid-state Nuclear Magnetic Resonance spectroscopy were recorded on a Ascend 600 MHz NMR spectrometer (Bruker, Germany). In detail, the electrolyte is prepared by using deuterium oxide (D_2_O) as solvent and 600 µL of the electrolyte was added to an NMR tube. UV-vis spectra were recorded on a Perkin Elmer Lambda 950 UV/Vis Spectrophotometer.

1.3 Electrochemical measurements in rotating disk electrode

For the Ag rotating disk electrode (RDE) tests, the diameter of the disk is 5 mm. The scan rate of CV tested on RDE set was 10 mV s^–1^. The electrolyte comprised of 0.1 M electrolytes with and without 5 mM EDTA, respectively. Before LSV testing, the electrolyte was saturated with N_2_ gas (99.99%) or CO_2_ gas (99.99%). Ag/AgCl electrode (saturated KCl) and platinum wire were used as reference electrode and counter electrode, respectively.

1.4 Electrocatalytic tests in an aqueous H-type cell

The Ag foil (Alfa Aesar, 0.127 mm thickness, 99.99%, metals basis), Zn foil (Alfa Aesar, 0.1 mm thickness, 99.994%), Sn foil (Alfa Aesar, 0.127 mm thickness, 99.99%), and Cu foil (Alfa Aesar, 0.127 mm thickness, 99.99%) were cut and ultrasonicated in acetone (HPLC, Ourchem), isopropanol (Alfa Aesar, 99.99%), and deionized water consecutively for 10 min each, and then rinsed with deionized water before electrochemical tests.

For the Ag NPs (Macklin, 99.99%, 60-120 nm), Pd/C (Alfa Aesar, 5% on carbon powder), Cu NPs (Macklin, 99.99%, 60-100 nm), and Bi NPs (Alfa Aesar, 99.99%), the electrocatalyst ink was prepared by dispersing catalyst (10 mg) in a mixture containing 960 μL of isopropanol and 40 μL 5 wt. % Nafion solution, followed by ultrasonication for 1 hour. Then, 100 μL of the catalyst ink was dripped onto carbon paper (Toray, TGP-H-060, area = 1 cm^2^). The electrolyte comprised of 0.5 M KHCO_3_ and different mole concentration of EDTA, which were dissolved in deionized water (PH = 7.2). Before each electrochemical test, the electrolyte was saturated with CO_2_ gas (99.99%).

All the electrochemical tests were performed though a potentiostat (DH7002, Donghua Testing Technology Co., Ltd.). The electrolysis was performed in a two-compartment H cell separated by an ion exchange membrane (Sigma-Aldrich, Nafion 117). A platinum network and Ag/AgCl (saturated KCl) were used for the counter and reference electrodes, respectively. The electrolyte in the cathodic compartment was stirred at a rate of 500 r.p.m. during electrolysis. CO_2_ gas was delivered into the cathodic compartment at a rate of 10 standard cubic centimetres per minute (sccm). Electrode potentials were converted to the RHE scale using E (versus RHE) = E (versus Ag/AgCl) + 0.625 V. The RHE calibration was performed under practical condition^19^. Linear sweep voltammetry and cyclic voltammetry were performed at a scan rate of 50 mV s^–1^. To more accurately assess the voltage-current response of the catalytic system, the steady-state staircase voltammetry (SCV) tests were performed. The increment potential of each step was set as 20 mV and the potential step period was set as 30 s.

The gas products were analyzed by an on-line gas chromatography (GC9790Ⅱ, FULI Instrument, Inc.), which was equipped with both a thermal conductivity detector (TCD) and a flame ionization detector (FID). The Faradaic efficiencies of H_2_ and CO were calculated using equation: Faradaic efficiency (%) =100×nFc/Q, where n is the number of electrons used in producing the products, F is the Faradaic constant (96485 C mol^–1^), c is the amount of the product (in moles), and Q is the total charge for the reduction process. Liquid products were quantified with ^1^H NMR spectrometer (600 MHz).

1.5 Electrocatalytic tests in a GDE configuration cell

A three-electrode flow-type cell was used to investigate the high-rate CO_2_ electrolysis performance of different electrolyte systems. Ag NPs (Macklin, 99.9%, 60~120 nm) ink was air-brushed onto a GDE (Sigracet, 28 BC) as the cathode (catalyst loading of 0.3 mg cm^–2^). The geometric area of the electrode window was 1× 1 cm^2^. Ag/AgCl electrode (saturated KCl) and commercial Ni foam (0.5 mm thickness) were used as reference electrode and counter electrode, respectively. The cathode and anode chambers were separated by an anion exchange membrane (Fumasep, FAB-PK-130). 1 M KHCO_3_ without and with 5 mM additives were used as catholyte, and 1 M KHCO_3_ was used as anolyte. The CO_2_ flow rate was 20 sccm controlled by a mass flow controller, and the flow rate of electrolyte was stabilized at 10 mL min^−1^. For electrocatalytic tests in the GDE set-up, a chronopotentiometry technique was used for 30 min.

1.6 Electrochemical Impedance Spectroscopy

Electrochemical impedance measurements were performed in an RDE cell fed with CO_2_-saturated 0.1 M KHCO_3_ electrolytes in the presence and absence of 5 mM EDTA. All impedance measurements were performed over a frequency range from 0.1 to 10^4.5^ Hz, and AC amplitude was set as 10 mV. We used the equivalent circuit of **Figure 2b** to simulate the experimental data points. The resulting Nyquist plots were analyzed with ZView2 software, where R_s_ is the solution resistance and R_ct_ is the charge transfer resistance. CPE is the constant phase element. The capacitive effects of EDL were modeled using a constant phase element (CPE) to offset the influence of the distributed time constant. The impedance of CPE is Y_0_^–1^(jꞷ)^–n^, where Y_0_ is the CPE coefficient, which is proportional to the double layer capacitance, and the exponent, n, is in the range of 0 ≤ n ≤ 1, which is directly related to the “frequency dispersion”^1-3^. The respective values of the circuit elements are presented in the **Tables S1** and **S2**.

1.7 In Situ ATR-SEIRAS

A polycrystalline Au nanofilm was deposited chemically onto a Si ATR-IR prism. Then Ag NPs (Macklin, 99.9%, 60~120 nm) were coated onto Au nanofilm as the working electrode. A platinum wire and Ag/AgCl (saturated KCl) were used for the counter and reference electrodes, respectively. ATR-SEIRAS experiments were conducted with ThermoFisher Nicolet IS20 spectrometer equipped with a liquid nitrogen-cooled MCT detector and a Si attenuated total reflection (UATR) accessory was used to collect the spectra. The spectral resolution was set to 8 cm^-1^. The data was collected with ~ 10 s resolution per spectrum and measured simultaneously by chronoamperometry technique from OCP (~ 0.6 V_RHE_) to –1.2 V_RHE_. Before the spectra were collected, the work electrodes were pre-reduced by cyclic voltammetry (0.2 to –0.7 V_RHE_, 20 mV s^–1^, 20 cycles) to keep the catalyst in a relatively stable state. Reference spectra for the SEIRAS measurements were recorded at OCP in CO_2_-saturated 0.5 M KHCO_3_ electrolytes with and without 1 mM EDTA. In order to ensure the reliability of the data, the two systems were tested using the same prism, specifically after the KHCO_3_ system was tested, EDTA was added to the electrolyte for testing.

1.8 In Situ Raman

Raman spectroscopy measurements were obtained using a LabRAM HR Evolution Raman spectrometer (HORIBA Scientific) equipped with a ×50 long working distance objective and a 532 nm laser. Raman frequency was calibrated to 520 nm by a Si wafer during each experiment. The laser was focused onto the surface of the Ag NPs (Macklin, 99.9%, 60~120 nm) electrode and the signal acquisition time for each spectrum was 10 s with an accumulation time of 1. The data was measured by chronoamperometry technique from open circuit potential (~ 0.62 V) to –0.9 V_RHE_. The measurements were conducted using a custom-made three-electrode spectroelectrochemical cell with a quartz window, in which the Ag NPs catalyst, Ag/AgCl (saturated KCl), and platinum wire were used as working, reference, and counter electrodes, respectively. The CO_2_-saturated 0.5 M KHCO_3_ electrolyte was pumped into the cell at a rate of 5 mL min^–1^ using a peristaltic pump. In situ Raman was applied to probe the adsorption of additive molecules on the electrode surface.

1.9 Materials characterization

The XRD patterns were taken with a Rigaku D/MAX 2550 diffractometer. XPS (Escalab 250Xi) was conducted using an Al Kα source, all data were corrected using the C 1s peak at 284.8 eV as an internal standard. SEM images were obtained with the Hitachi S-4800 scanning electron microscope.

***2.* Computational methods**

2.1 MD simulation.

To analyze the solvation structure of K^+^ ion when the EDTA is introduced into the KCl solution, the molecular dynamics (MD) were performed using LAMMPS. In MD simulations, the forced field parameters were obtained from OPLS-AA force fields. The SPC/E water model is employed for H_2_O molecule^4,5^ and the electrostatic interactions were computed using the particle-mesh Ewald (PME) method^6^. The size of box is 10 × 10 × 10 nm^3^, and periodic boundary conditions were set in all three directions. The cell was constructed with 33000 H_2_O, 300 K^+^, 300 Cl^-^, 6 EDTA. A cut-off length of 1.0 nm was used in the calculation of electrostatic interactions and non-electrostatic interactions in real space. The temperature was maintained at 300 K using Nose−Hoover thermostat with a damping constant of 200 fs. The integration time step was 2 fs, and the data are saved every 2 ps. The first run lasting 20 ns on the NPT ensemble is used to obtain a reasonable relaxation structure. Another 20 ns long run on the NVT ensemble follows to equilibrate the system, in which the final 2 ns was used to gather the statistical quantities.

2.2 DFT calculation.

Surface analysis was calculated by density function theory (DFT) calculation. Based on the optimized structures from MD simulations, the molecules were fully optimized by the Gaussian 09 program^7^ with the B3LYP /6-311+G (d, p)^8,9^, including empirical dispersion corrections DFT-D3 method^10,11^. The implicit universal solvation model based on Solute Electron Density (SMD) with a dielectric constant of water was employed to investigate the influence of solvent. The electrostatic potential (ESP) was analyzed by the Multiwfn program^12^ and visualized via the VMD package^13^. All basis sets are obtained from the Basis Set Exchange library.

2.3 AIMD simulation.

The ab initio molecular dynamics in this work were carried out with a periodic slab model using the Vienna ab initio simulation program (VASP)^14^, and the Perdew–Burke–Ernzerhof (PBE) exchange-correlation functional was chosen^15^. The surface slab of Ag(111) with a periodic four-layer model (20.0 Å×11.6 Å×30.0 Å) was geometrically relaxed. The Brillouin zone was sampled at the Γ-point. The projector-augmented wave (PAW) method^16,17^ was utilized to describe the electron–ion interactions and the cut-off energy for the plane-wave basis set was 400 eV. The convergence criterion for the electronic self-consistent iteration was set to be 10^-5^ eV. To illustrate the long-range dispersion interactions between the adsorbates and catalysts, we employed the D3 correction method as described by Grimme et al.^11^ To investigate the influence of EDTA, two pre-equilibrated solvent configurations (78 H_2_O molecules, 78 H_2_O + 1 EDTA molecules) were inserted on top of the Ag surface respectively and underwent AIMD for 4.0 ps to equilibrate the two systems, where the EDTA molecule was parallelly located at the second solvation layer. Then, we inserted a K^+^ ion in a cavity of the first solvation layer close to the surface, subsequently removing a hydrogen from a water molecule in the outermost water layer (K-H_2_O system) and a hydrogen from the EDTA molecule (K-H_2_O-EDTA system) to keep the charge balance, respectively. To optimize the H-bond network of both systems, we carried out AIMD simulations for a total of 3.0 ps with a 1 fs time step in a canonical NVT (particle number, volume and temperature) ensemble at 300 K regulated by a Nosé–Hoover thermostat. The final 0.5 ps of both systems was used to analyze influence of EDTA on the H-bond network. Furthermore, we added a CO_2_ to the resulting K-H_2_O and K-H_2_O-EDTA systems proximal to the Ag surface and the K^+^ ion. Then, the obtained K-H_2_O-CO_2_ and K-H_2_O-EDTA-CO_2_ systems underwent AIMD for 4.5 and 6.0 ps, respectively, and were considered as the initial configurations for modeling the subsequent CO_2_ reduction and HER processes.

2.4 Enhanced Sampling.

Constrained ab initio molecular dynamics (cAIMD) simulations with a slow-growth (SG) sampling approach as implemented in VASP are performed to evaluate the kinetic barriers of proton transfer during both CO_2_ reduction and HER^18,19^. It defines a suitable collective variable (CV, namely ξ) as the reaction coordinate to evaluate the linear transformation from an initial state to a final state. In the SG sampling, the transformation step ∂ξ is chosen as 0.0004 $Å$ for each cAIMD step. The work required to perform the transformation from initial to final states can be computed as:

$$W_{\text{initial​​ → final}}\text{​​​ =​​​​}\int_{\text{ξ}_{\text{initial}}}^{\text{ξ}_{\text{final}}} \text{​​}\left( \frac{\text{∂F}}{\text{∂ξ}} \right)\text{·}\dot{\xi}\text{d​t}$$

where *F* is the time-dependent free energy, $\frac{\partial F}{\partial\xi}$ can be computed using the blue-moon ensemble with the SHAKE algorithm^20^, and $\dot{\xi}$ is the transformation velocity from the initial state to the final state. The reaction barriers and reaction energies are obtained by computing the free energy profiles after thermodynamic integrations^21,22^.


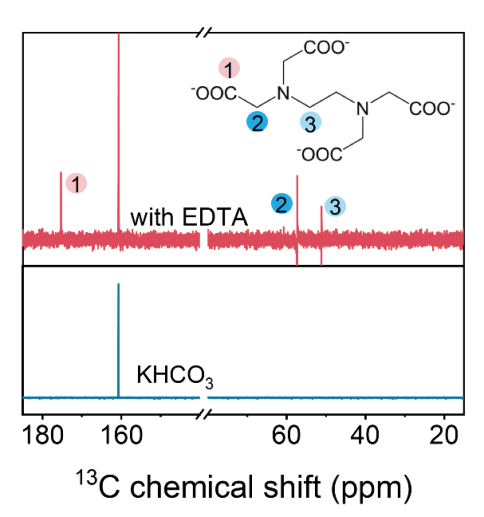


**Figure S1.** ^13^C-NMR spectra of KHCO_3_ electrolytes with and without 5 mM EDTA.


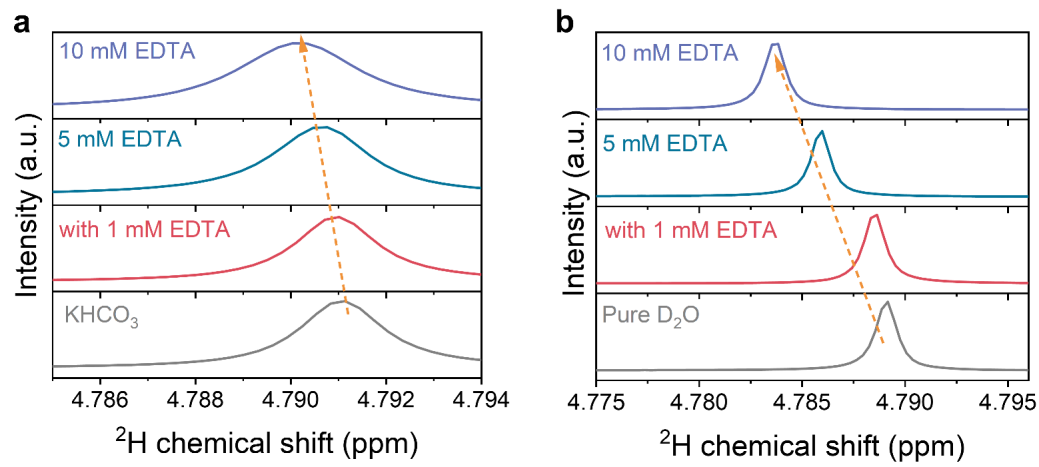


**Figure S2.** **a**, ^2^H-NMR spectra of the KHCO_3_ electrolytes without and with different concertation of EDTA. **b**, ^2^H-NMR spectra of the pure D_2_O without and with different concertation of EDTA.


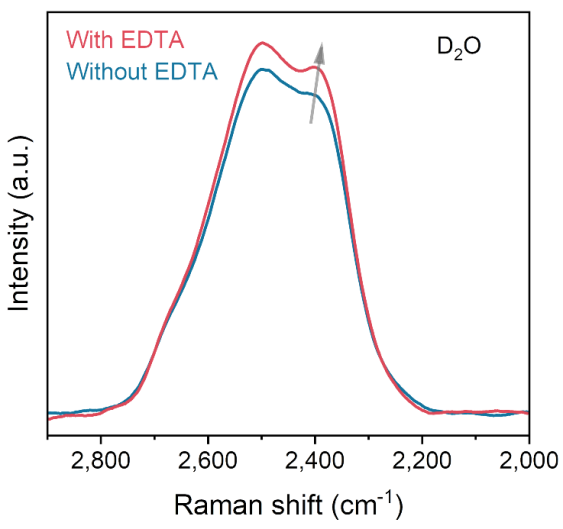


**Figure S3.** Raman spectra of *ν*-OH mode of 0.5 M KHCO_3_ electrolyte with and without 5 mM EDTA. The electrolyte is prepared using D_2_O.

The prominent signal near 2380 cm^-1^ indicates the presence of EDTA-H_2_O hydrogen-bonding in the electrolyte.


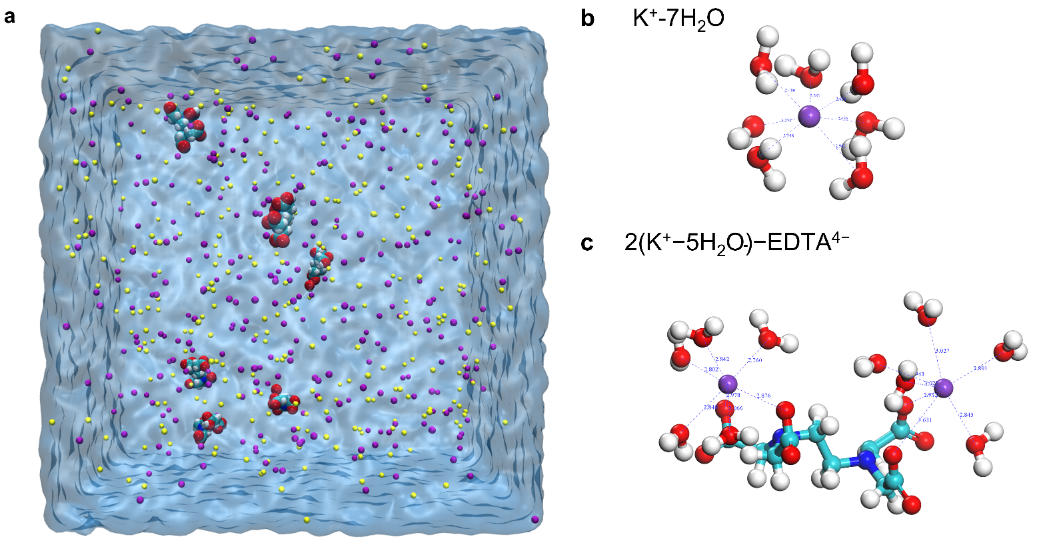


**Figure S4. a,** Snapshot of the KCl-EDTA system from the MD simulation. **b**, Local distribution of water molecules near the K^+^ ion. **c**, Solvation structure of the K^+^ ion affected by the EDTA.


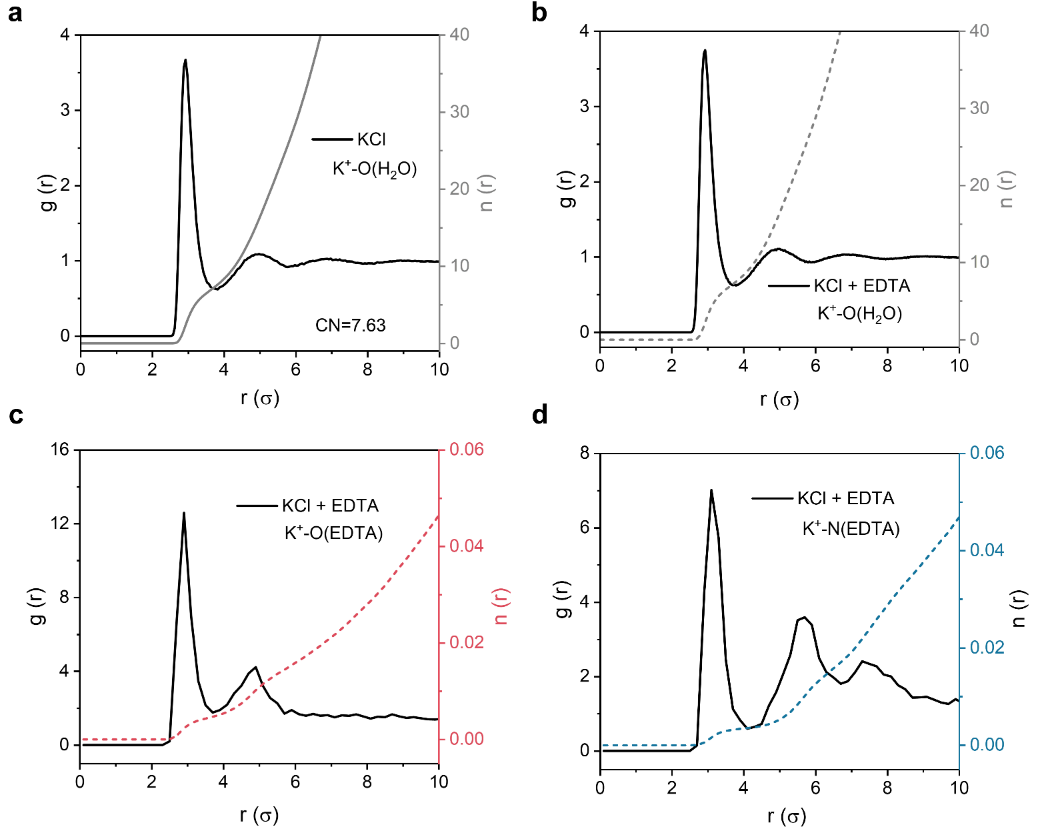


**Figure S5.** Radial distribution functions (RDFs) for (**a**) K^+^-O (H_2_O) in the KCl system, (**b**) K^+^-O (H_2_O), (**c**) K^+^-O (EDTA) and (**d**) K^+^-N (EDTA) in the KCl + EDTA system.


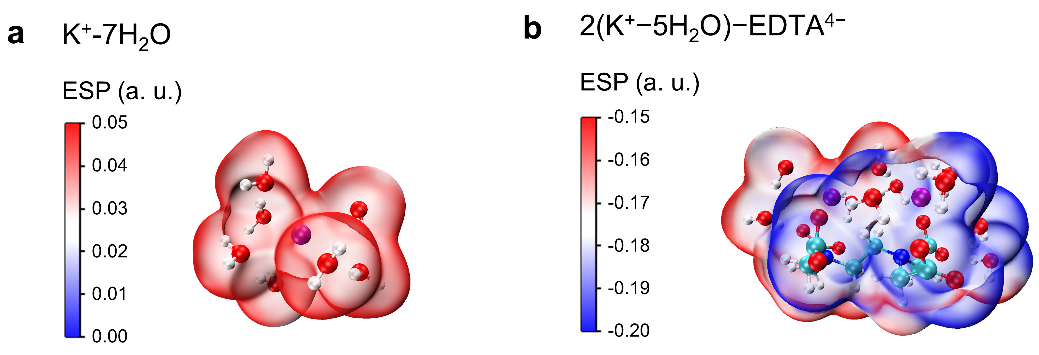


**Figure S6.** Electrostatic potential mapping for K^+^-7H_2_O (**a**) and 2(K^+^-5H_2_O)-EDTA^4-^ (**b**) solvation structures.


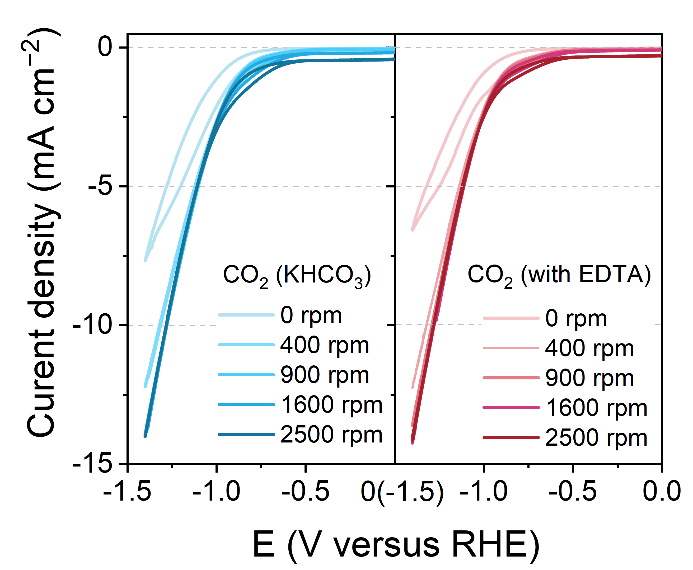


**Figure S7.** CV curves in KHCO_3_ electrolytes with and without 5 mM EDTA at a scan rate of 10 mV s^−1^ under CO_2_-saturated conditions.


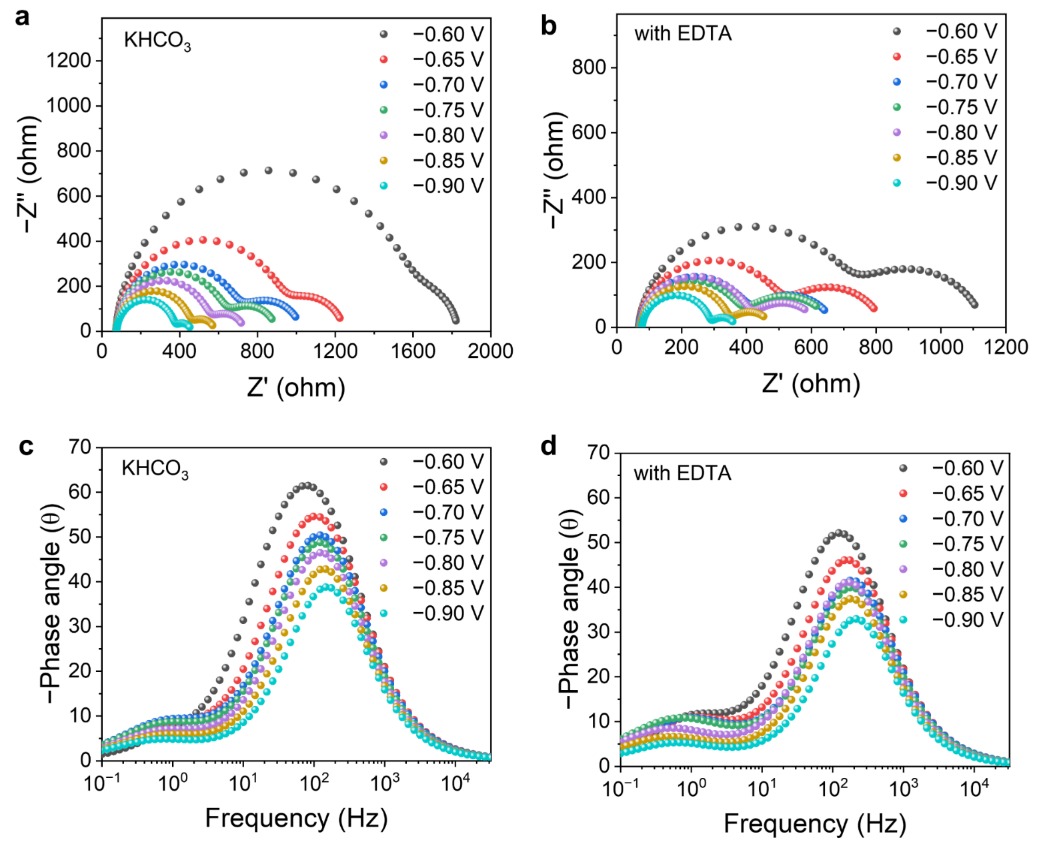


**Figure S8. a,** Nyquist plots of KHCO_3_ system measured from –0.6 to –0.9 V_RHE_. **b**, Nyquist plots of EDTA-containing system measured from –0.6 to –0.9 V_RHE_. **c**, Bode plots of KHCO_3_ system measured from –0.6 to –0.9 V_RHE_. **d**, Bode plots of EDTA-containing system measured from –0.6 to –0.9 V_RHE_.


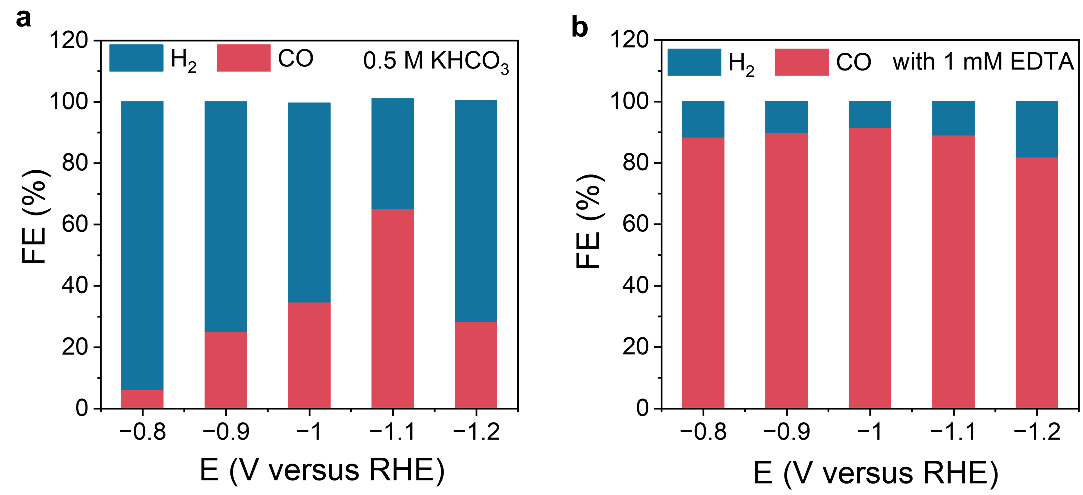


**Figure S9. a,** Faradaic efficiency of Ag foil electrode in 0.5 M KHCO_3_ from –0.8 to –1.2 V_RHE_. **b,** Faradaic efficiency of Ag foil electrode in 0.5 M KHCO_3_ with 1 mM EDTA from –0.8 to –1.2 V_RHE_. Electrochemical tests were conducted in an aqueous H-type cell.


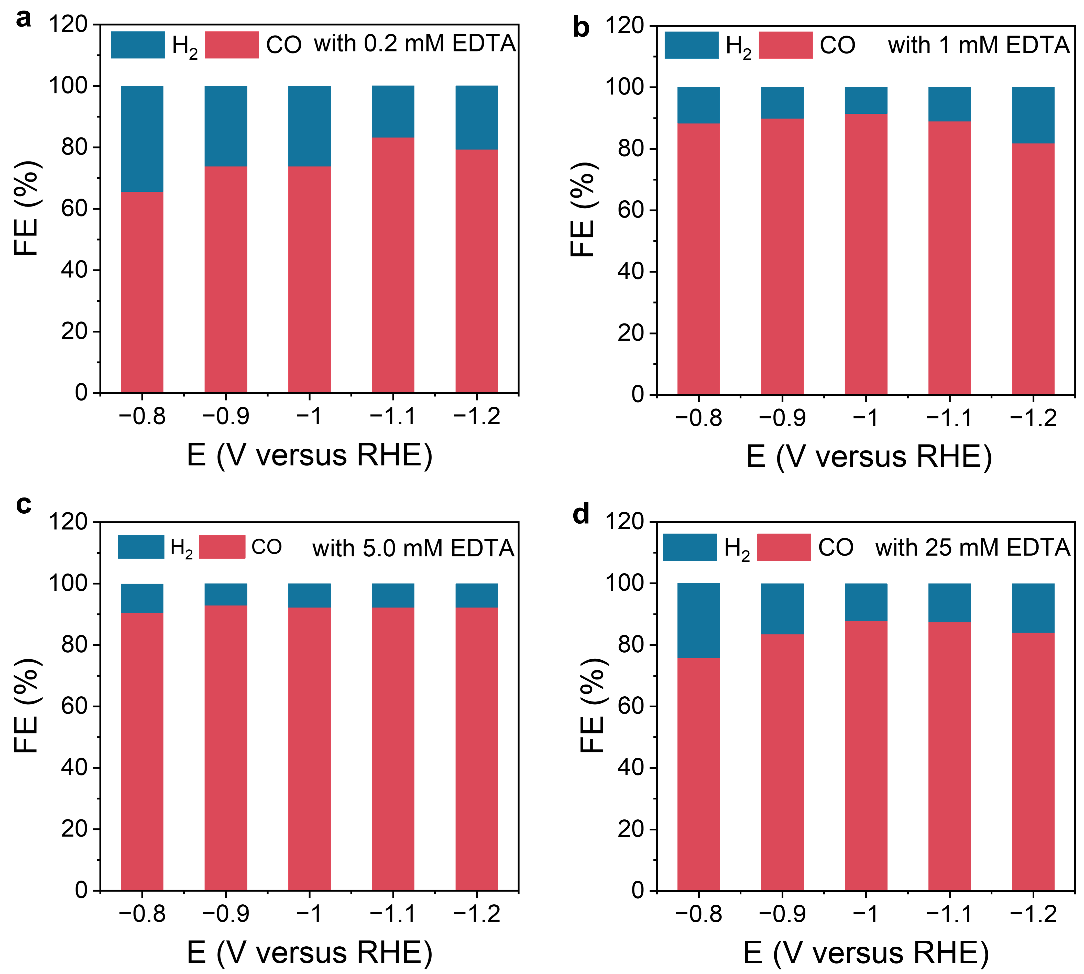


**Figure S10.** Faradaic efficiency of Ag foil electrode in 0.5 M KHCO_3_ with 0.2 mM EDTA (**a**), 1 mM EDTA (**b**), 5 mM EDTA (**c**), 25 mM EDTA (**d**), from –0.8 to –1.2 V_RHE_. Electrochemical tests were conducted in an aqueous H-type cell.


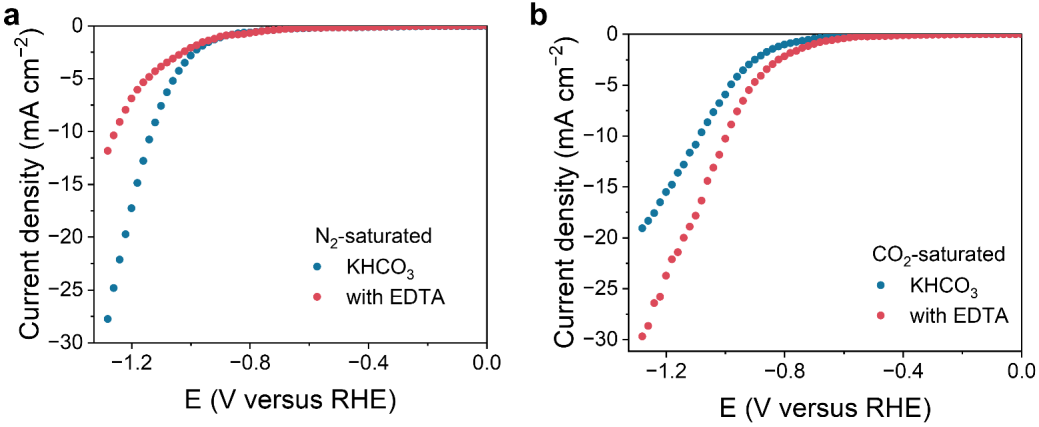


**Figure S11**. SCV tests in KHCO_3_ electrolytes without and with 5 mM EDTA under N_2_- (**a**) and CO_2_- (**b**) saturated conditions in H-type cell.


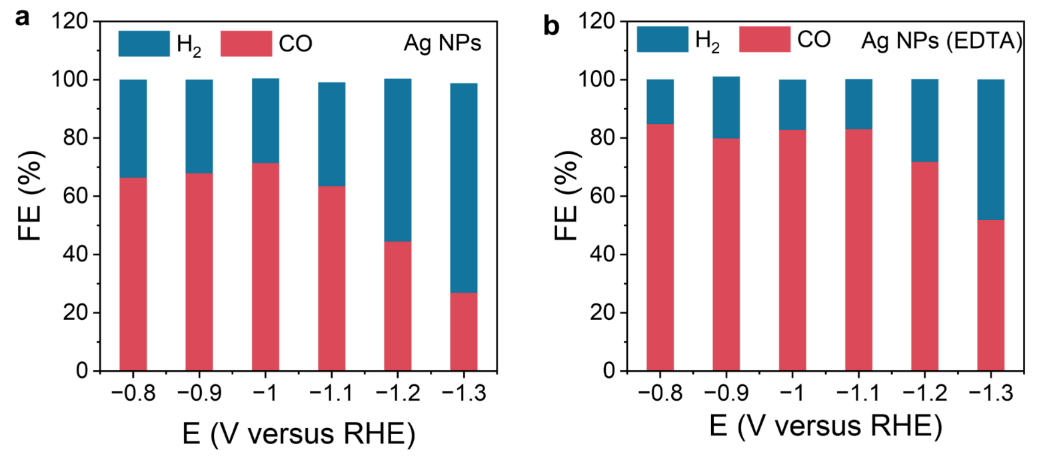


**Figure S12. a,** Faradaic efficiency of Ag NPs electrode in 0.5 M KHCO_3_ from –0.8 to –1.2 V_RHE_. **b,** Faradaic efficiency of Ag NPs electrode in 0.5 M KHCO_3_ with 1 mM EDTA from –0.8 to –1.2 V_RHE_. Electrochemical tests were conducted in an aqueous H-type cell.


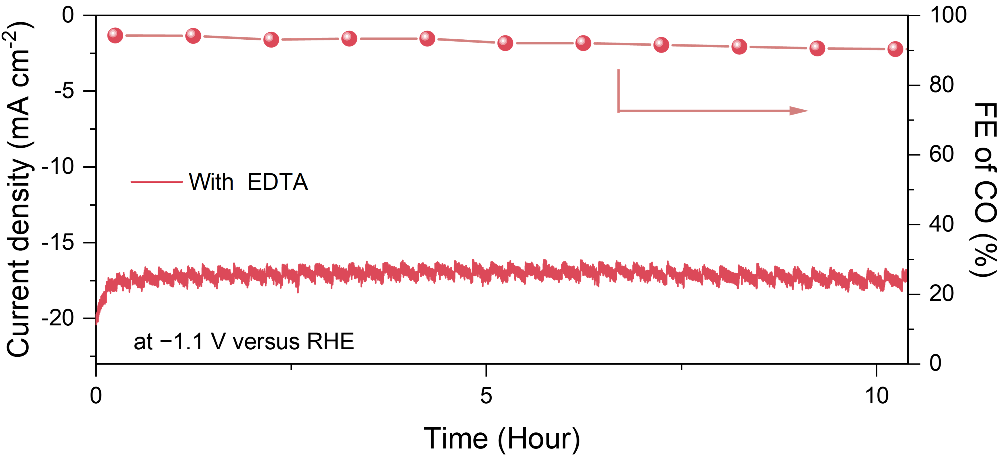


**Figure S13.** Long-term catalytic performance of Ag foil electrode in 0.5 M KHCO_3_ with 1 mM EDTA in an aqueous H-type cell.


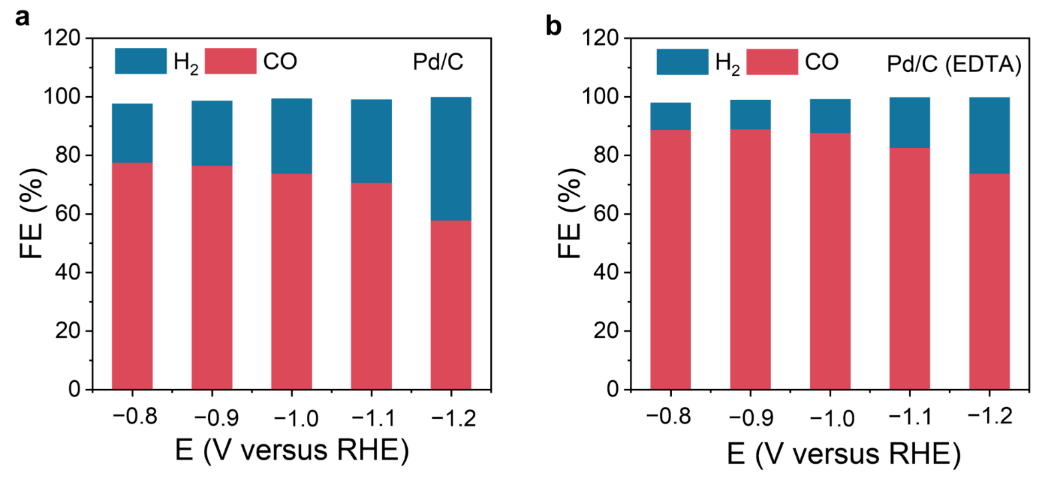


**Figure S14. a,** Faradaic efficiency of Pd/C electrode in 0.5 M KHCO_3_ from –0.8 to –1.2 V_RHE_. **b,** Faradaic efficiency of Pd/C electrode in 0.5 M KHCO_3_ with 1 mM EDTA from –0.8 to –1.2 V_RHE_. Electrochemical tests were conducted in an aqueous H-type cell.


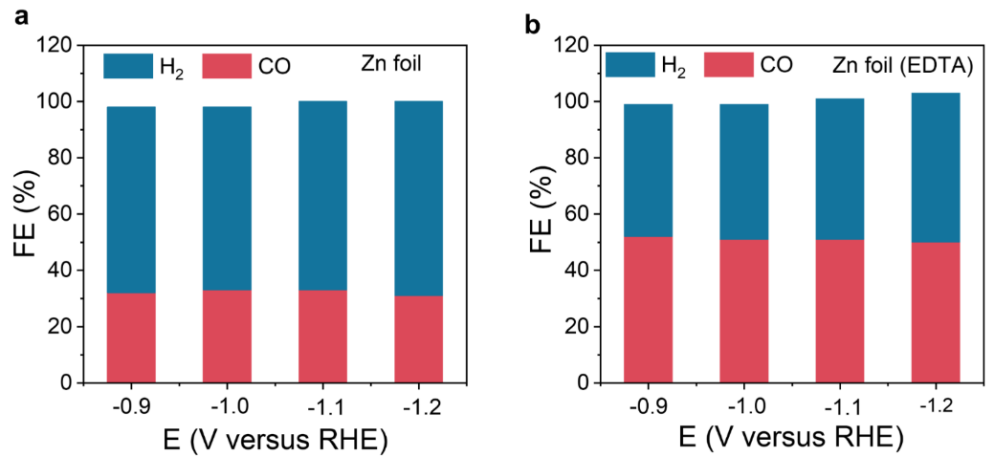


**Figure S15. a,** Faradaic efficiency of Zn foil electrode in 0.5 M KHCO_3_ from –0.9 to –1.2 V_RHE_. **b,** Faradaic efficiency of Zn foil electrode in 0.5 M KHCO_3_ with 1 mM EDTA from –0.9 to –1.2 V_RHE_. Electrochemical tests were conducted in an aqueous H-type cell.


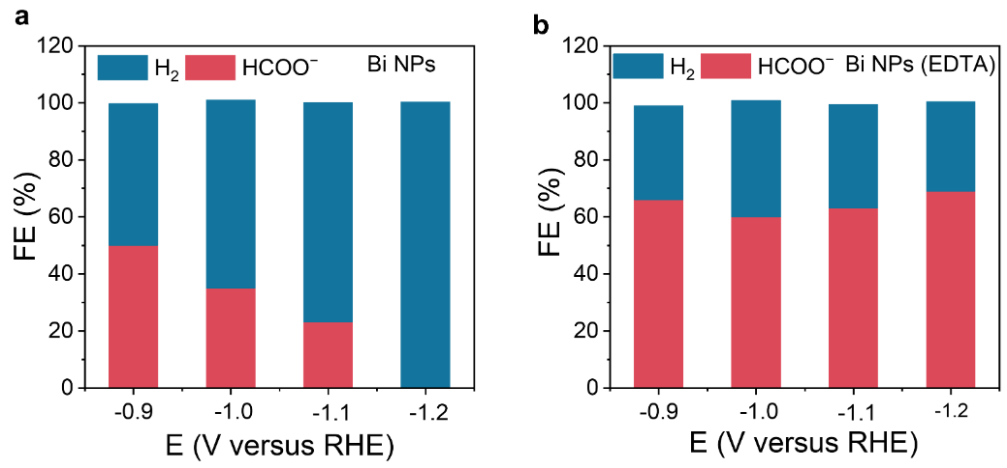


**Figure S16. a,** Faradaic efficiency of Bi NPs electrode in 0.5 M KHCO_3_ from –0.9 to –1.2 V_RHE_. **b,** Faradaic efficiency of Bi NPs electrode in 0.5 M KHCO_3_ with 1 mM EDTA from –0.9 to –1.2 V_RHE_. Electrochemical tests were conducted in an aqueous H-type cell.


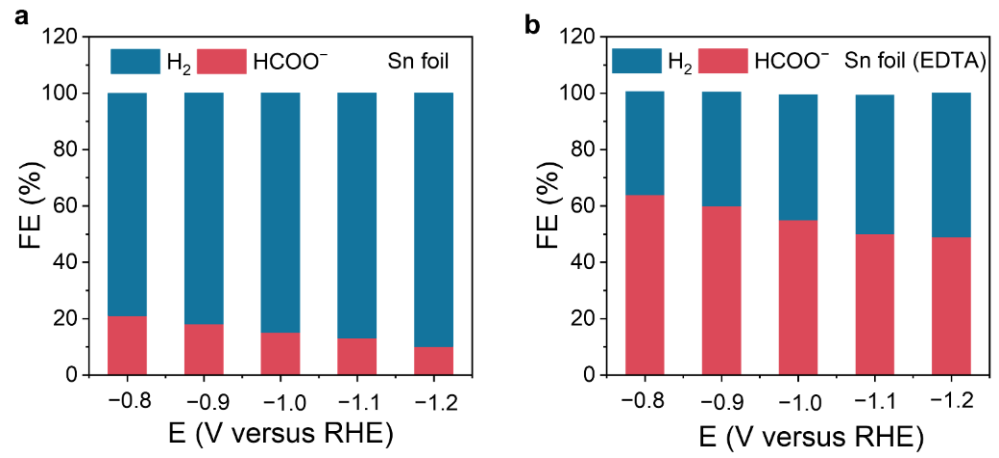


**Figure S17. a,** Faradaic efficiency of Sn foil electrode in 0.5 M KHCO_3_ from –0.8 to –1.2 V_RHE_. **b,** Faradaic efficiency of Sn foil electrode in 0.5 M KHCO_3_ with 1 mM EDTA from –0.8 to –1.2 V_RHE_. Electrochemical tests were conducted in an aqueous H-type cell.


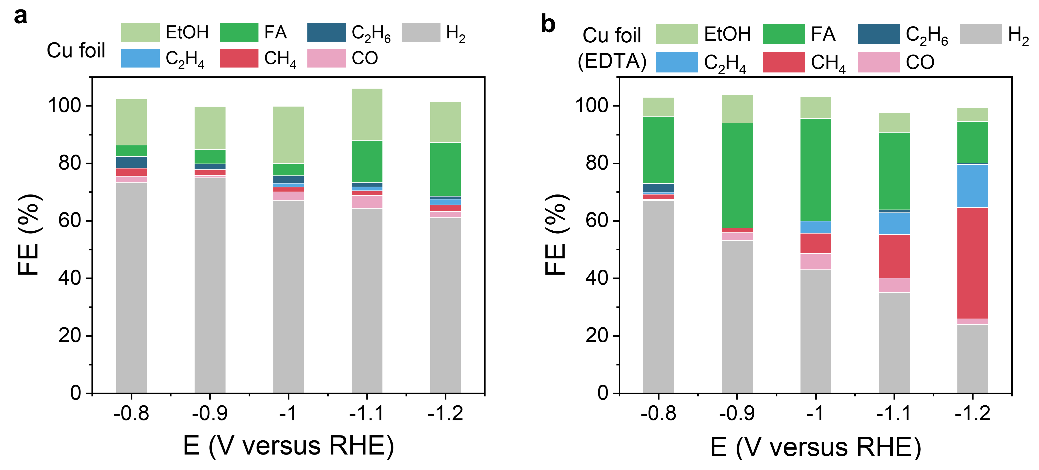


**Figure S18. a,** Faradaic efficiency of Cu foil electrode in 0.1 M KHCO_3_ from –0.8 to –1.2 V_RHE_. **b,** Faradaic efficiency of Cu foil electrode in 0.1 M KHCO_3_ with 1 mM EDTA from –0.8 to –1.2 V_RHE_. Electrochemical tests were conducted in an aqueous H-type cell.


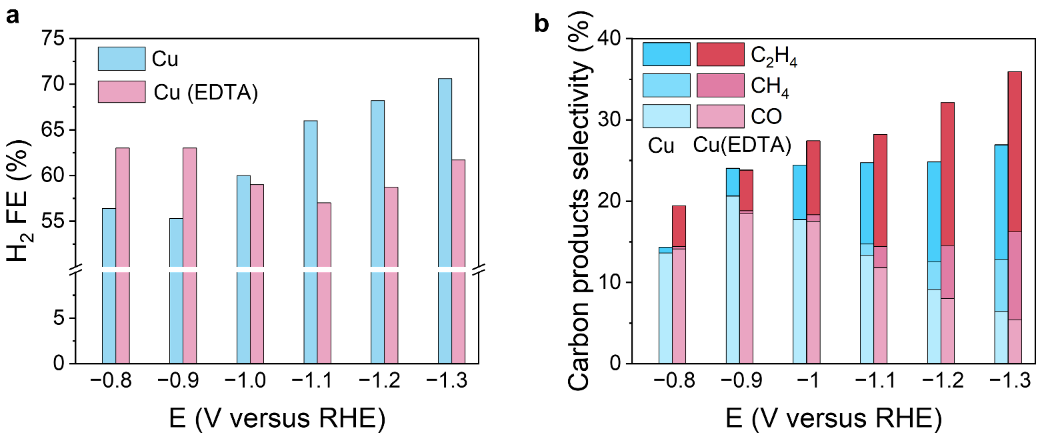


**Figure S19.** Faradaic efficiency of H_2_ (**a**) and carbon-based product selectivity (**b**) of Cu NPs electrode in 0.1 M KHCO_3_ with and without 1 mM EDTA from –0.8 to –1.3 V_RHE_. Electrochemical tests were conducted in an aqueous H-type cell.


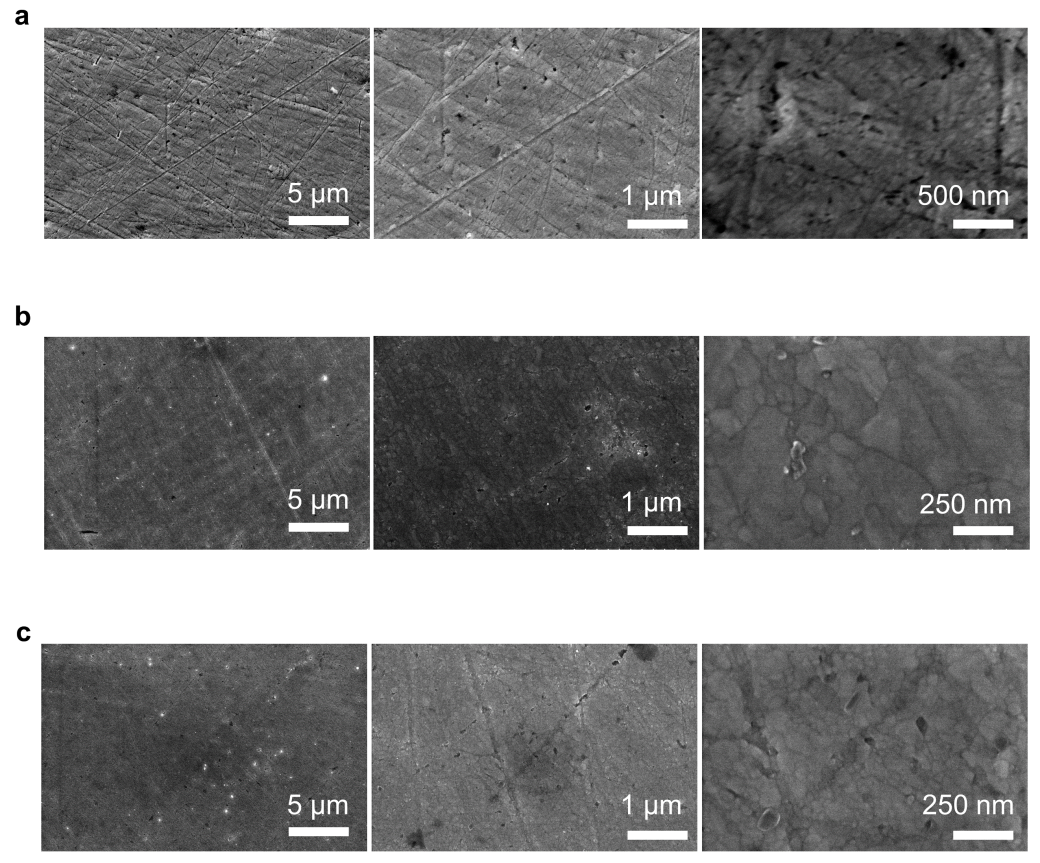


**Figure S20. a,** SEM images of Ag foil electrode before CO_2_ electrolysis. **b**, SEM images of Ag foil electrode after CO_2_ electrolysis in 0.5 M KHCO_3_ with 1 mM EDTA. **c**, SEM images of Ag foil electrode after CO_2_ electrolysis in 0.5 M KHCO_3_.


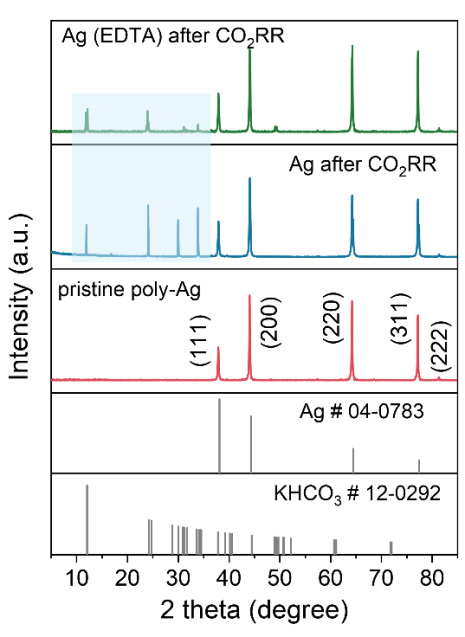


**Figure S21.** XRD patterns of Ag foil electrode before and after CO_2_ reduction reaction (CO_2_RR) in two electrolyte systems.

The highlighted areas reflect the amount of bicarbonate remaining on the surface of the Ag foil after testing. Compared to the blank system, the EDTA system exhibits lower KHCO_3_ signal intensity, suggesting that the EDTA-reshaped interfacial environment may slow down salt deposition. This is conducive to the improvement of electrolytic stability.


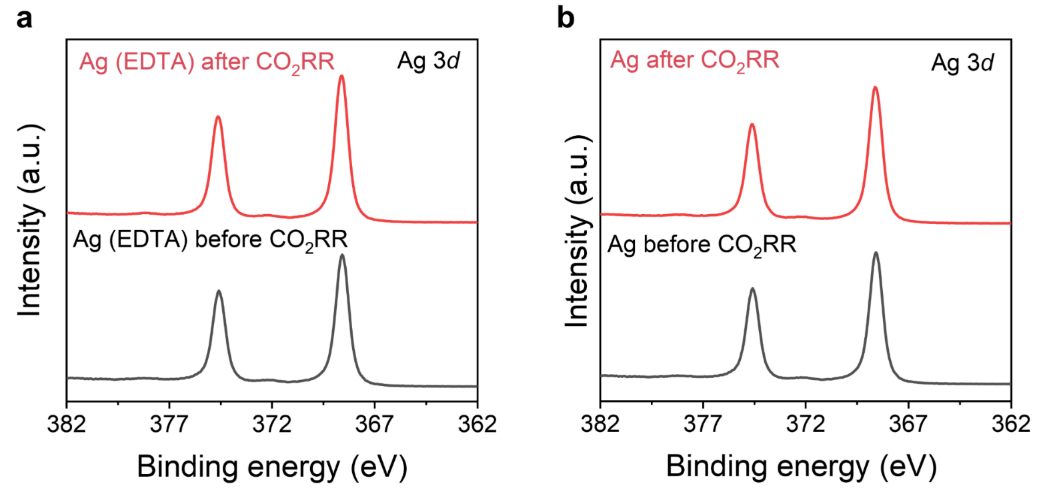


**Figure S22. a,** XPS spectral of Ag foil electrode before and after CO_2_ reduction reaction (CO_2_RR) in 0.5 M KHCO_3_ with 1 mM EDTA. **b**, XPS spectral of Ag foil electrode before and after CO_2_ reduction reaction (CO_2_RR) in 0.5 M KHCO_3_.


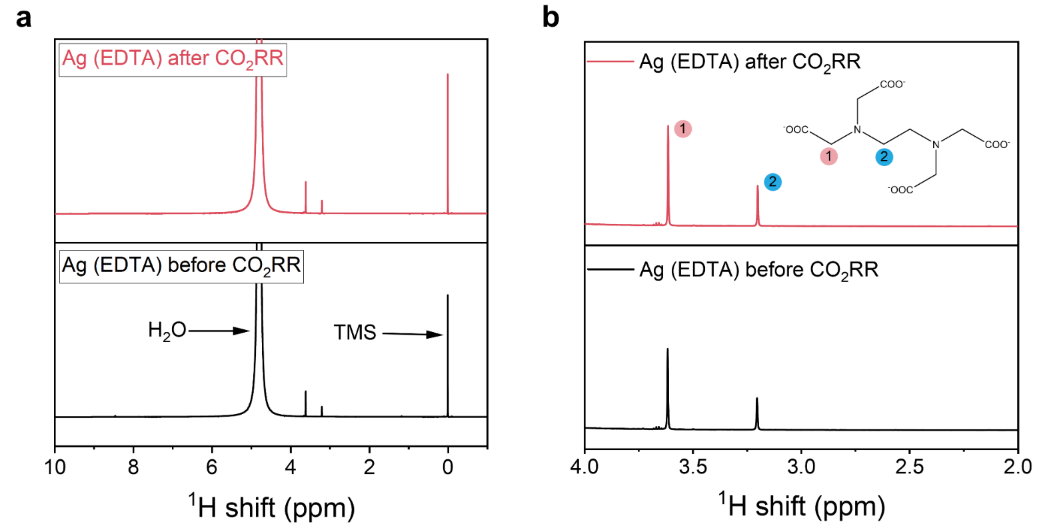


**Figure S23. a,** ^1^H-NMR spectra of 0.5 M KHCO_3_ with 1 mM EDTA before and after CO_2_ reduction reaction (CO_2_RR). **b**, Local enlargement of Fig. a between 4 to 2 ppm.


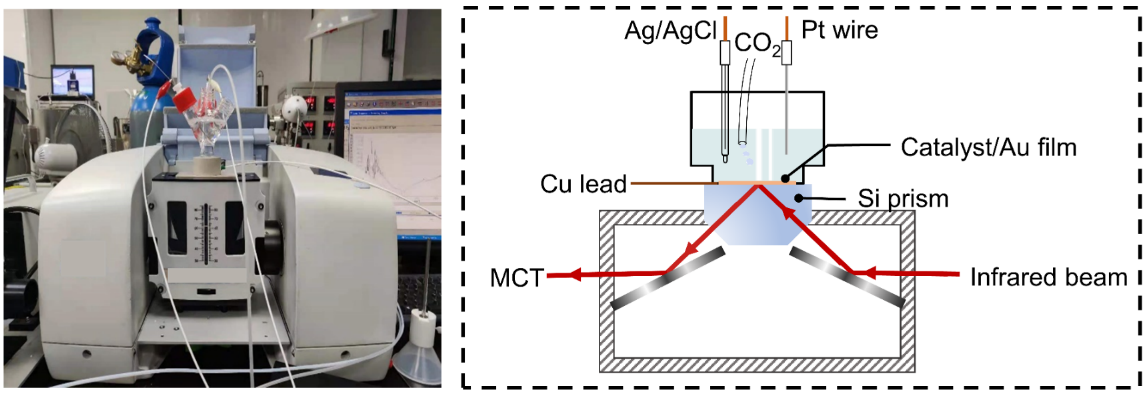


**Figure S24.** Photograph and schematic of in situ ATR-SEIRAS.


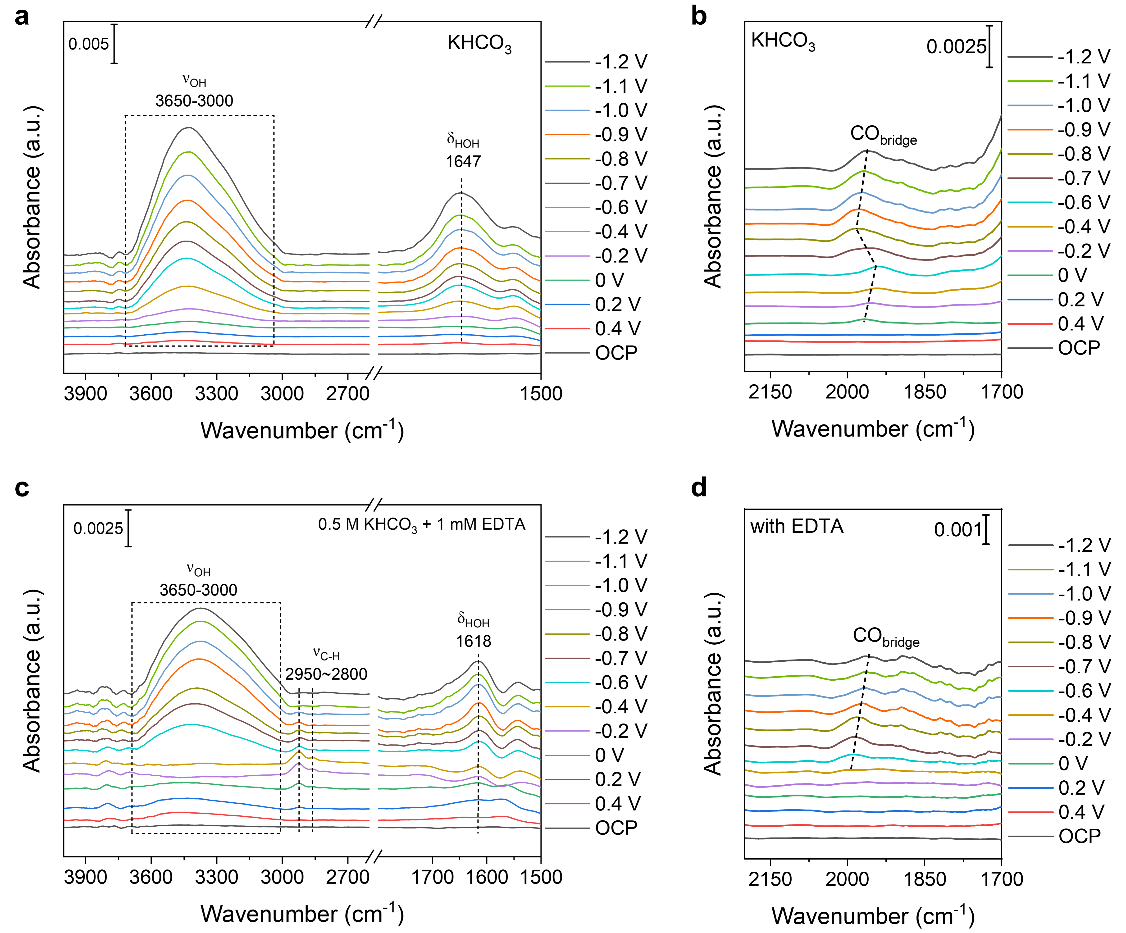


**Figure S25. a**,**b,** In situ ATR-SEIRAS spectra under various potentials for the KHCO_3_ electrolyte. **c**,**d**, In situ ATR-SEIRAS spectra under various potentials for the KHCO_3_ electrolyte with 1 mM EDTA.


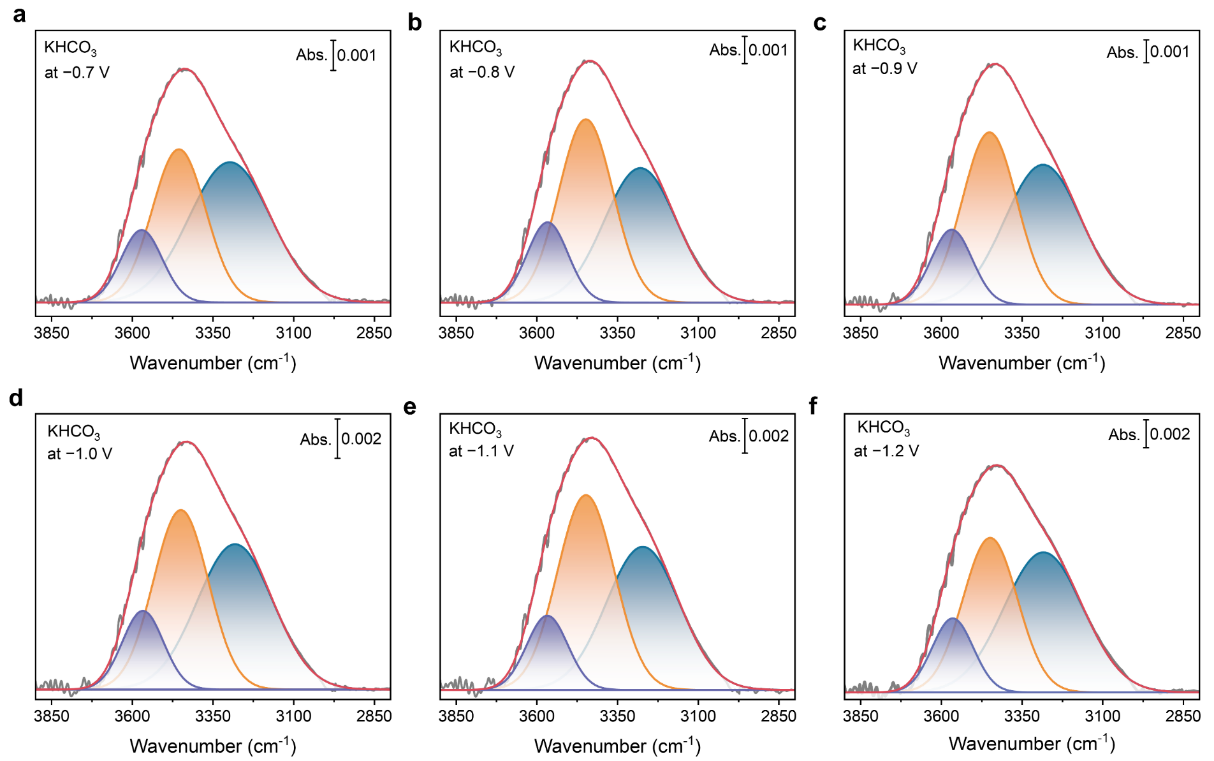


**Figure S26.** Deconvolution of the ν-OH peak in 0.5 M KHCO_3_ electrolyte at −0.7 (**a**), −0.8 (**b**), −0.9 (**c**), −1.0 (**d**), −1.1 (**e**) and −1.2 (**f**) V_RHE_.


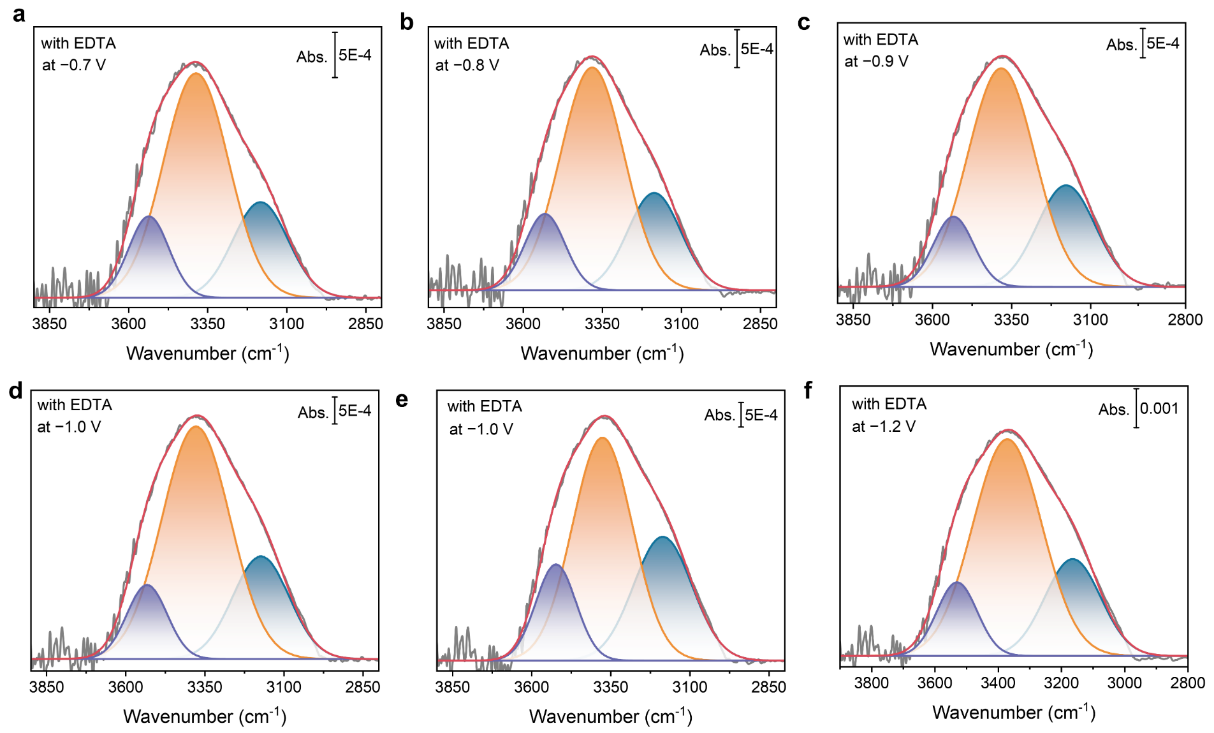


**Figure S27.** Deconvolution of the ν-OH peak in 0.5 M KHCO_3_ electrolyte with 1 mM EDTA at −0.7 (**a**), −0.8 (**b**), −0.9 (**c**), −1.0 (**d**), −1.1 (**e**) and −1.2 (**f**) V_RHE_.


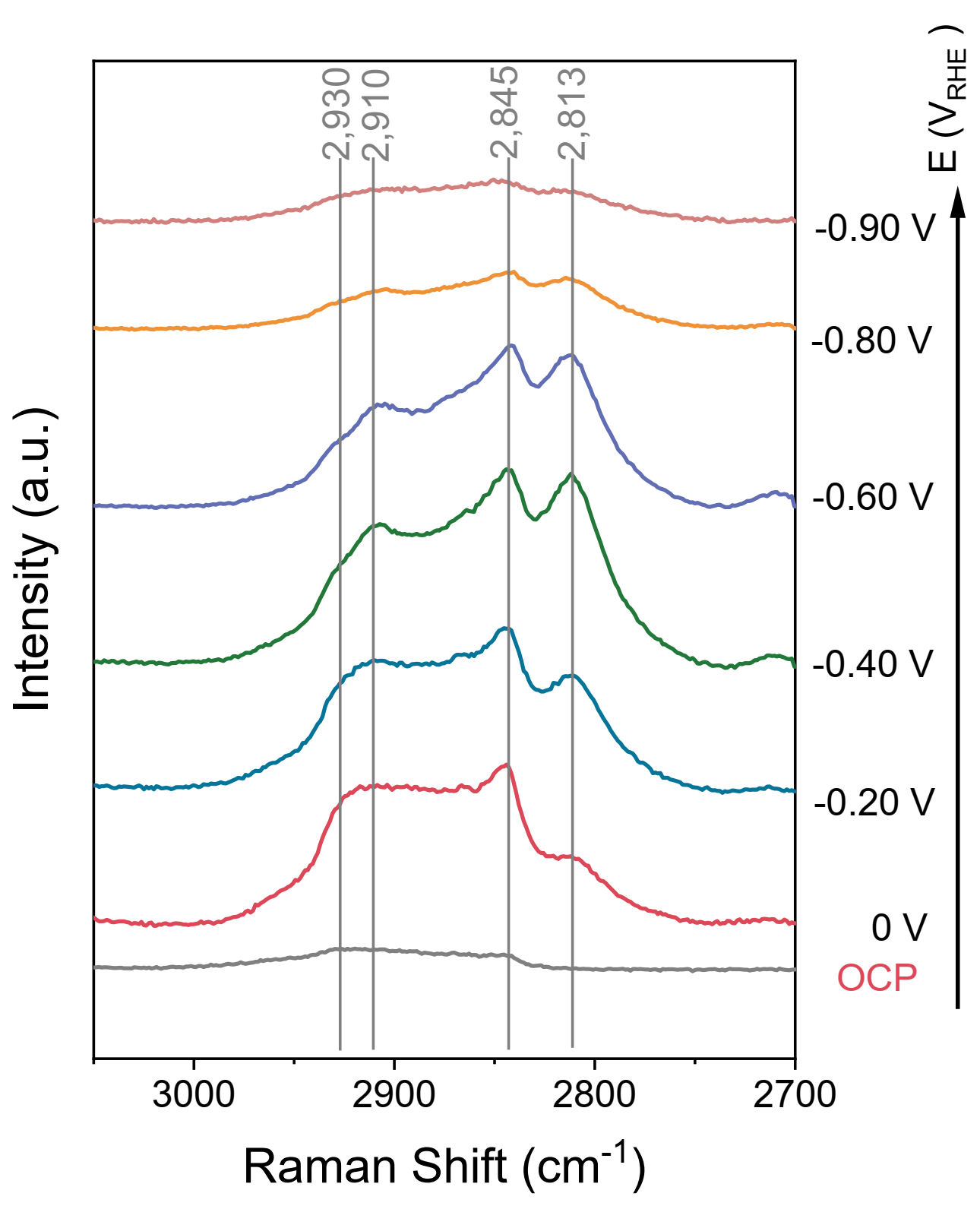


**Figure S28.** In situ Raman spectra of the C–H stretching mode of EDTA molecules at Ag surfaces measured in CO_2_-saturated 0.5 M KHCO_3_ with 1 mM EDTA.


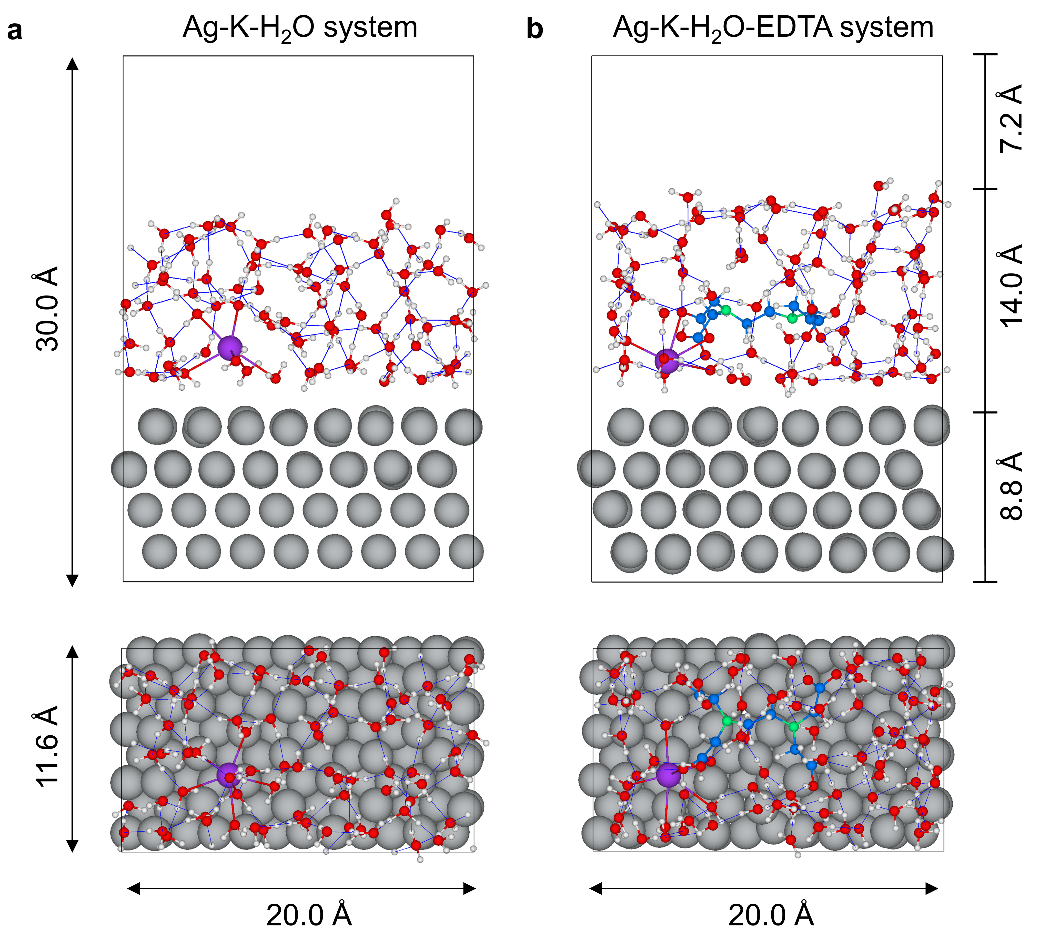


**Figure S29.** Input models of hydrogen-bond analysis for the AIMD calculations carried out on an Ag(111) supercell (20.0 Å × 11.6 Å × 30.0 Å) with K-H_2_O (**a**) and K-H_2_O-EDTA systems (**b**).


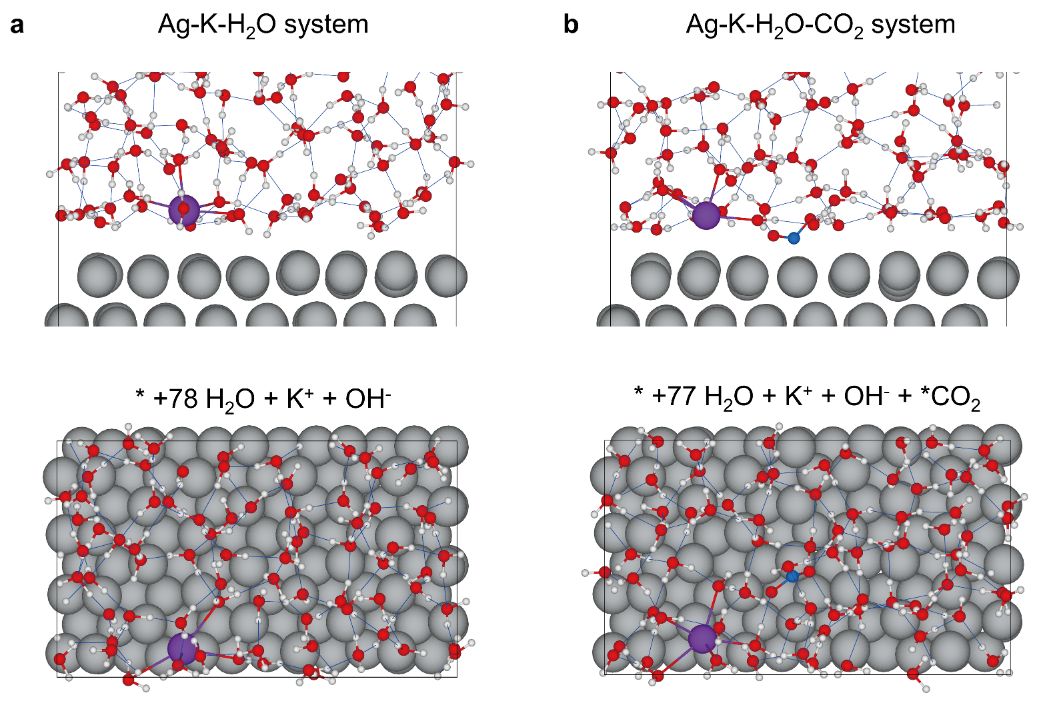


**Figure S30.** **a**, Optimized result of Ag-K-H_2_O system after undergoing AIMD for 3 ps. **b**, Input models for constrained AIMD calculations carried out on an Ag(111) supercell (20.0 Å × 11.6 Å × 30.0 Å) with inserting a CO_2_ molecule into the resulting Ag-K-H_2_O system.


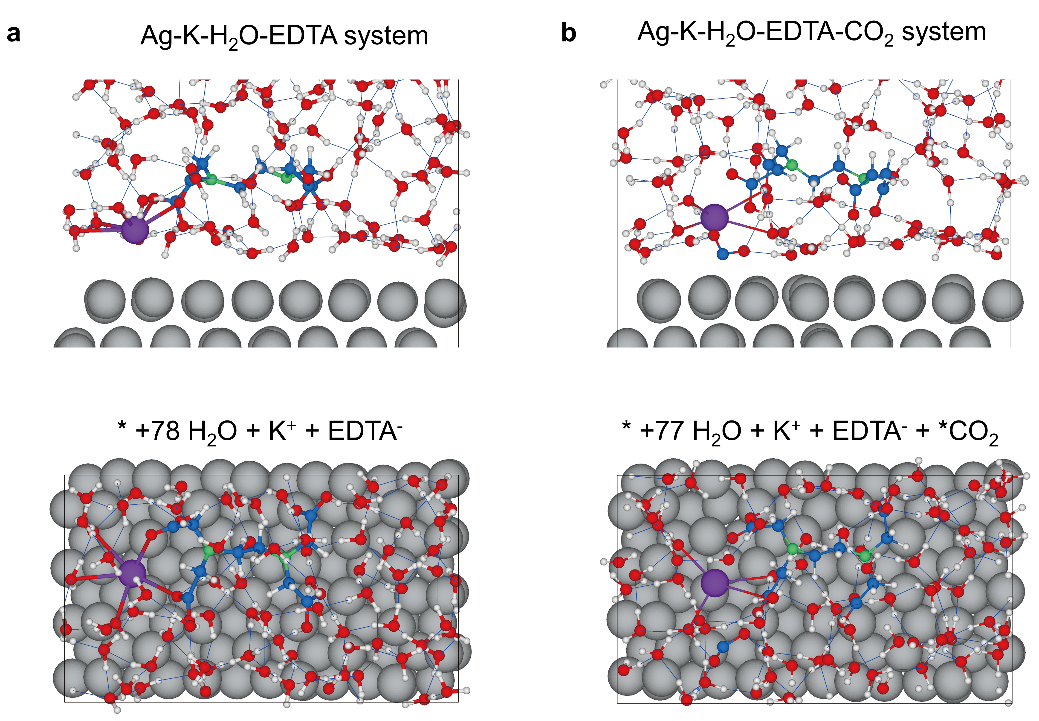


**Figure S31.** **a**, Optimized result of Ag-K-H_2_O-EDTA system after undergoing AIMD for 3 ps. **b**, Input models for constrained AIMD calculations carried out on an Ag(111) supercell (20.0 Å × 11.6 Å × 30.0 Å) with inserting a CO_2_ molecule into the resulting Ag-K-H_2_O-EDTA system.


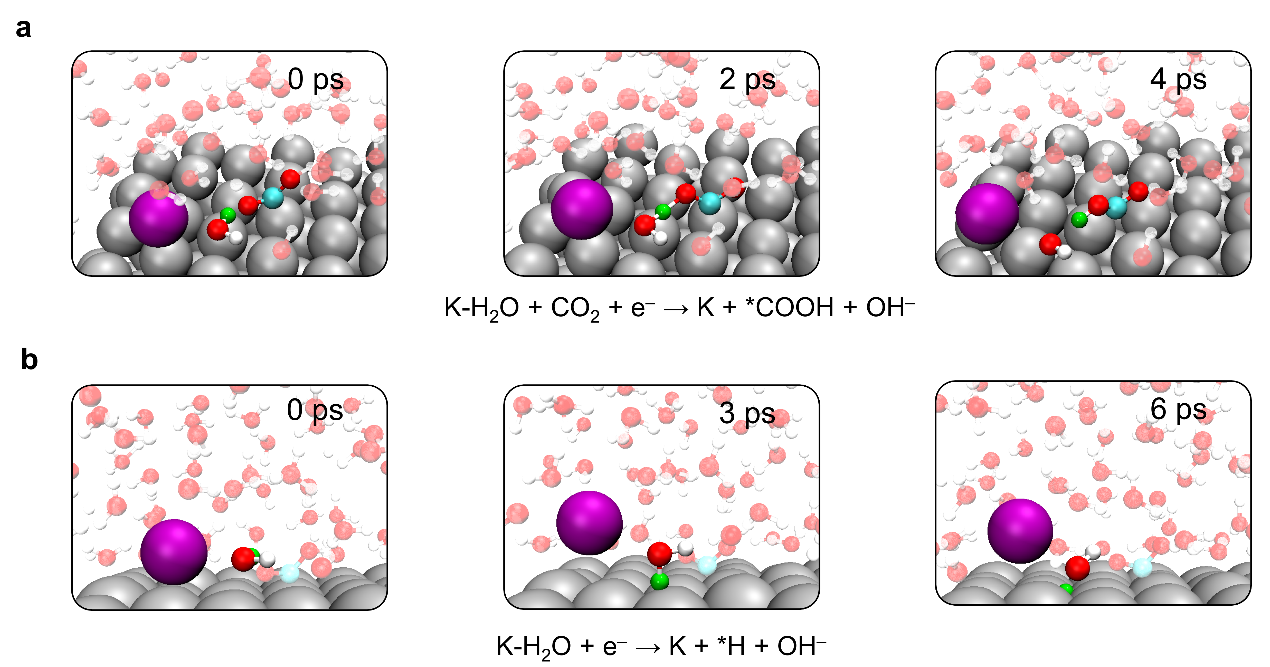


**Figure S32.** Representive snapshots for initial, intermediate and final states for (**a**) the CO_2_ reduction from *CO_2_ to *COOH and (**b**) the water dissociation from H_2_O to *H.


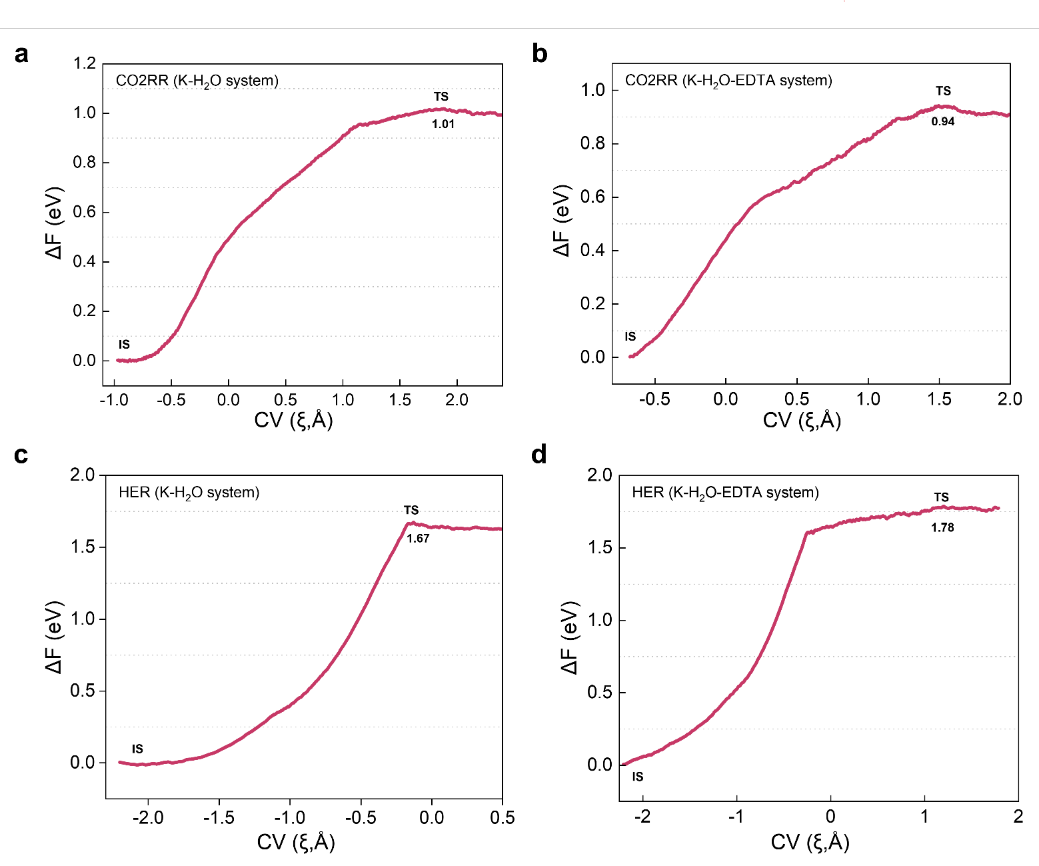


**Figure S33.** Free energy profiles of (**a**, **b**) the *CO_2_-to-*COOH step and (**c**, **d**) the H_2_O-to-*H step via cAIMD simulations in the K-H_2_O and K-H_2_O-EDTA systems, respectively.


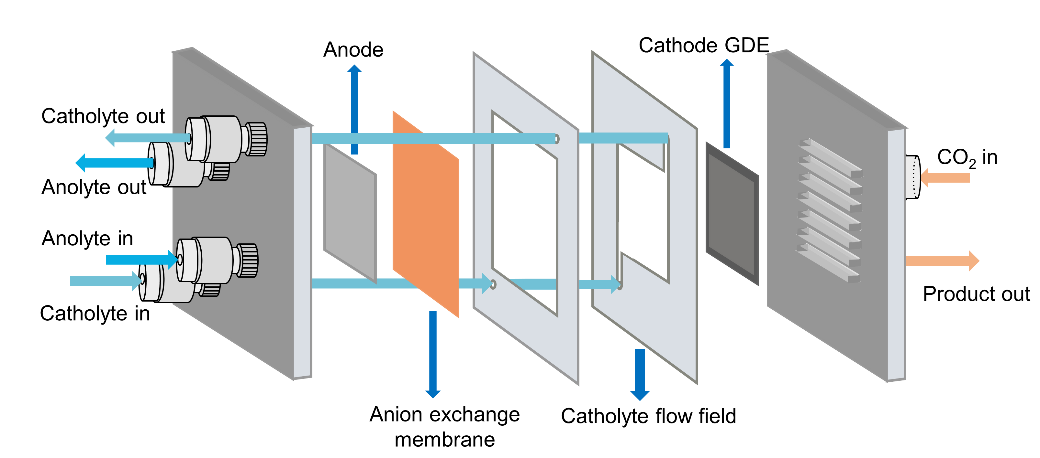


**Figure S34.** Schematics of flow-type cell.


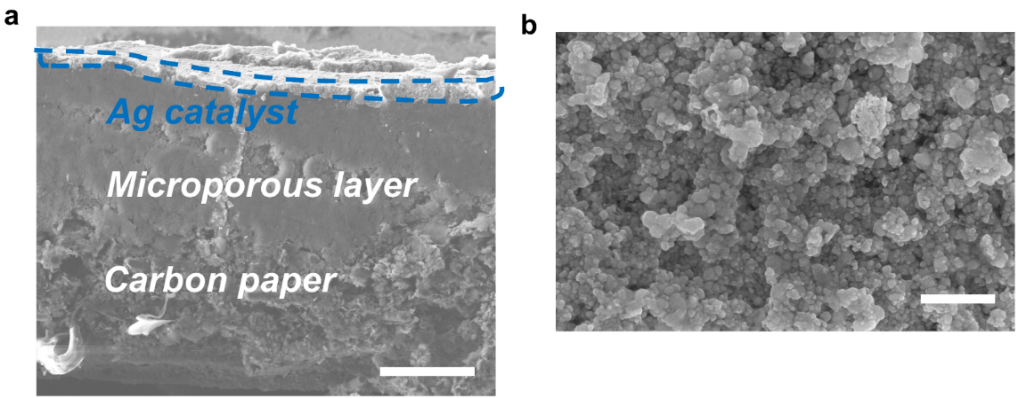


**Figure S35. a,** Cross-sectional SEM image of a gas diffusion electrode, which consists of carbon paper (top), a microporous layer (middle) and catalyst layer (bottom) (scale bar: 70 μm). **b**, SEM images of Ag NPs assembled on a gas diffusion electrode (GDE) (scale bar: 1 μm).


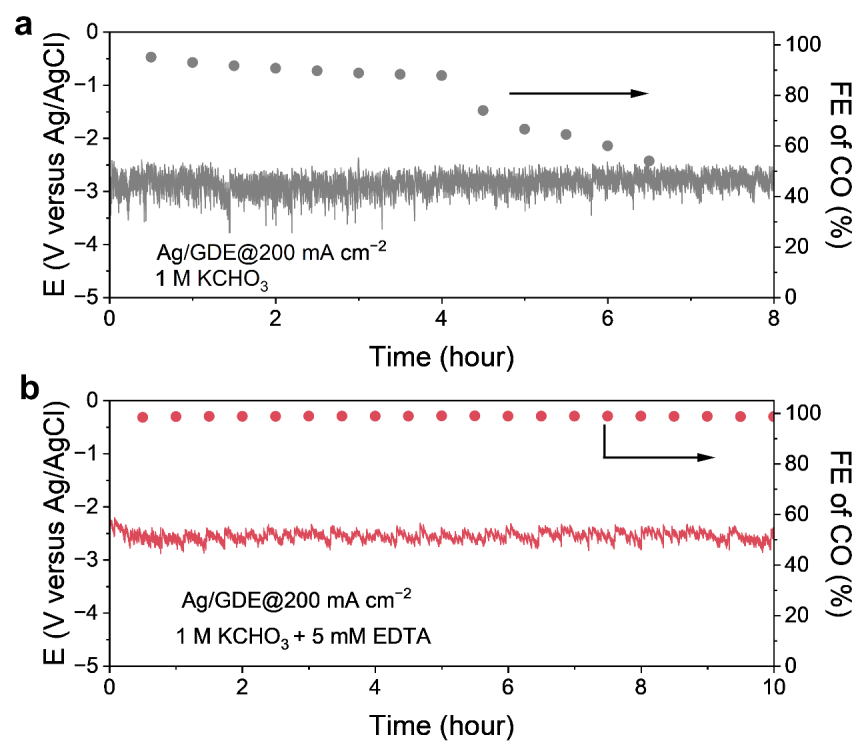


**Figure S36.** Long-term catalytic performance of Ag/GDE electrode in 1 M KHCO_3_ without (**a**) and with 5 mM EDTA (**b**) in flow-type cell.

The EDTA-containing system delivers a good stability in the 10 h test. And it showed less current fluctuation, which also further indicates its more stable electrolytic process.


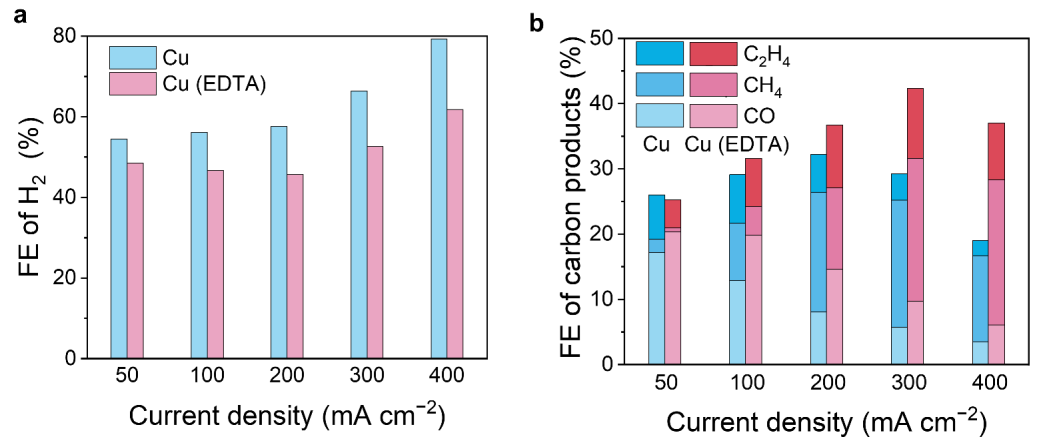


**Figure S37.** Faradaic efficiency of H_2_ (**a**) and carbon-based product selectivity (**b**) of Cu NPs electrode in 1 M KHCO_3_ with and without 5 mM EDTA from 50 to 400 mA cm^–2^. Electrochemical tests were conducted in flow-type cell.


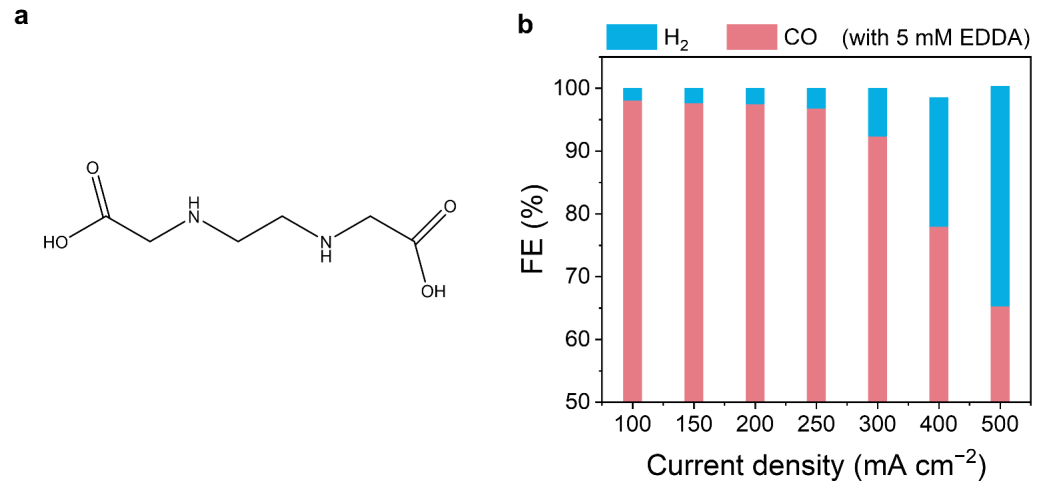


**Figure S38. a,** Structural formulae of ethylenediamine-N, N'-diacetic acid (EDDA). **b**, Faradaic efficiency of H_2_ and CO at various total current densities in 1 M KHCO_3_ electrolytes with 5 mM EDDA in flow-type cell.


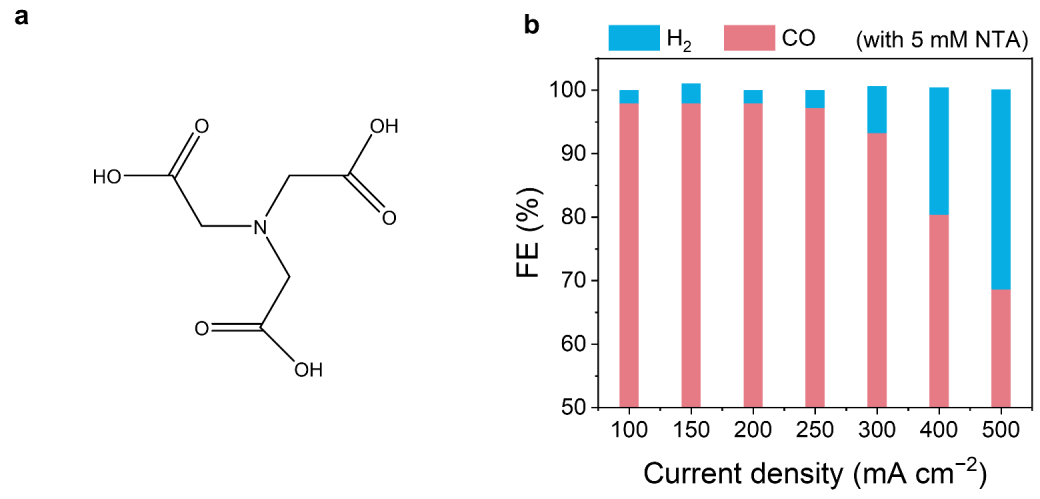


**Figure S39. a,** Structural formulae of nitrilotriacetic acid (NTA). **b**, Faradaic efficiency of H_2_ and CO at various total current densities in 1 M KHCO_3_ electrolytes with 5 mM NTA in flow-type cell.


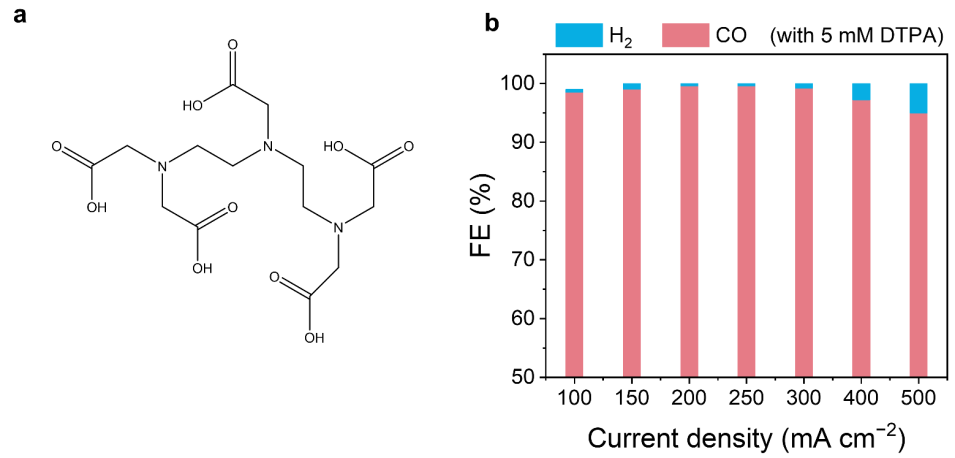


**Figure S40. a,** Structural formulae of diethylenetriaminepentaacetic acid (DTPA). **b**, Faradaic efficiency of H_2_ and CO at various total current densities in 1 M KHCO_3_ electrolytes with 5 mM DTPA in flow-type cell.


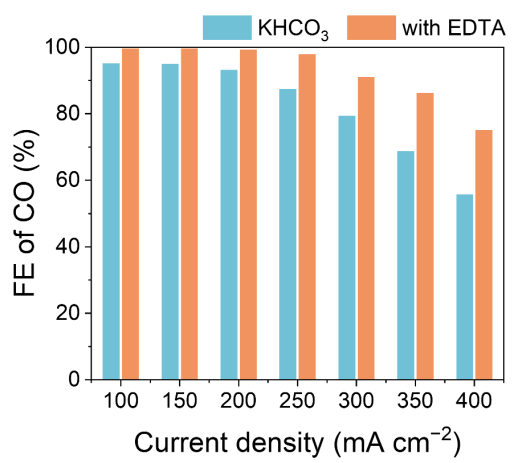


**Figure S41.** CO_2_ electrolysis performance in a 10 cm^2^ flow cell at applied current densities from 100 to 400 mA cm^–2^. The tests were carried out in the electrolyte of 1 M KHCO_3_ with and without 5 mM EDTA.


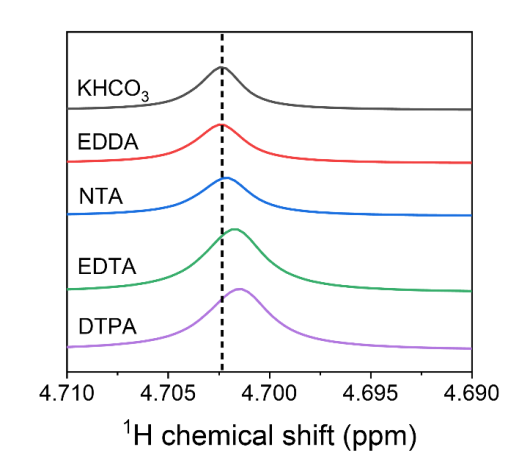


**Figure S42.** ^1^H-NMR spectra of the different additive molecules. The concentration of additives is 5 mM.

As shown in above figure, from EDDA to DTPA, as the number of carboxyl groups of the additive molecule increases, the shift degree of the water peak toward the higher field increases, resulting in a more significant disruption of H-bond network of H_2_O-H_2_O by the additive-H_2_O interaction.

**Table S1.** Simulated impedance of 0.1 M KHCO_3_ electrolyte with 5 mM EDTA system over Ag RDE electrode at different potentials.

| E (V versus RHE) | R_ct_  (Ω) | Y_0_/CPE  (μΩ^-1^·s^n^) | n |
| --- | --- | --- | --- |
| −0.60 | 677.4 | 9.2871 | 0.93945 |
| −0.65 | 450.8 | 9.3829 | 0.93743 |
| −0.70 | 341.8 | 9.8108 | 0.93324 |
| −0.75 | 315.7 | 10.197 | 0.93304 |
| −0.80 | 339.6 | 10.021 | 0.94238 |
| −0.85 | 272.1 | 9.7747 | 0.94968 |
| −0.90 | 211.9 | 9.6198 | 0.95288 |

**Table S2.** Simulated impedance of 0.1 M KHCO_3_ electrolyte system over Ag RDE electrode at different potentials.

| E (V versus RHE) | R_ct_  (Ω) | Y_0_/CPE  (μΩ^-1^·s^n^) | n |
| --- | --- | --- | --- |
| −0.60 | 1556 | 8.8524 | 0.94342 |
| −0.65 | 885.2 | 9.1202 | 0.9398 |
| −0.70 | 648.1 | 9.6273 | 0.93708 |
| −0.75 | 575.5 | 10.179 | 0.93736 |
| −0.80 | 488.7 | 10.577 | 0.9412 |
| −0.85 | 387.6 | 10.937 | 0.94221 |
| −0.90 | 303.9 | 11.15 | 0.94347 |

***3.* Supplementary References**

1. Bandarenka A S. Exploring the interfaces between metal electrodes and aqueous electrolytes with electrochemical impedance spectroscopy. *Analyst* 2013; **138**: 5540–54.

2. Wang S, Zhang J, Gharbi O *et al*. Electrochemical impedance spectroscopy. *Nat Rev Methods Primers* 2021; **1**: 41.

3. Khademi M, Barz D P J. Structure of the Electrical Double Layer Revisited: Electrode Capacitance in Aqueous Solutions. *Langmuir* 2020; **36**: 4250–60.

4. Berendsen H J C, Grigera J R, Straatsma T P. The missing term in effective pair potentials. *J Phys Chem* 1987; **91**: 6269–71.

5. Chatterjee S, Debenedetti P G, Stillinger F H *et al*. A computational investigation of thermodynamics, structure, dynamics and solvation behavior in modified water models. *J Chem Phys* 2008; **128**: 124511.

6. Essmann U, Perera L, Berkowitz M L *et al*. A smooth particle mesh Ewald method. *J Chem Phys* 1995; **103**: 8577–93.

7. Frisch M J, Trucks G W, Schlegel H B *et al*. Gaussian 09. Gaussian, Inc. Wallingford CT 2009.

8. Hehre W J, Ditchfield R, Pople J A. Self—Consistent Molecular Orbital Methods. XII. Further Extensions of Gaussian—Type Basis Sets for Use in Molecular Orbital Studies of Organic Molecules. *J Chem Phys* 2003; **56**: 2257–61.

9. Becke A D. Density‐functional thermochemistry. III. The role of exact exchange. *J Chem Phys* 1993; 98: 5648–52.

10. Grimme S, Ehrlich S, Goerigk L. Effect of the damping function in dispersion corrected density functional theory. *J Comput Chem* 2011; **32**: 1456–65.

11. Grimme S, Antony J, Ehrlich S *et al*. A consistent and accurate ab initio parametrization of density functional dispersion correction (DFT-D) for the 94 elements H-Pu. *J Chem Phys* 2010; **132**: 154104.

12. Lu T, Chen F. Multiwfn: A multifunctional wavefunction analyzer. *J Comput Chem* 2012; **33**: 580–92.

13. Humphrey W, Dalke A, Schulten K. VMD: Visual molecular dynamics. *J Mol Graph* 1996; **14**: 33–8.

14. Kresse G, Furthmüller J. Efficient iterative schemes for ab initio total-energy calculations using a plane-wave basis set. *Phys Rev B* 1996; **54**: 11169–86.

15. Perdew J P, Burke K, Ernzerhof M. Generalized Gradient Approximation Made Simple. *Phys Rev Lett* 1996; 77: 3865–68.

16. Kresse G, Joubert D. From ultrasoft pseudopotentials to the projector augmented-wave method. *Phys Rev B* 1999; **59**: 1758–75.

17. Blöchl P E. Projector augmented-wave method. *Phys Rev B* 1994; **50**: 17953–79.

18. Woo T K, Margl P M, Blöchl P E *et al*. A Combined Car−Parrinello QM/MM Implementation for ab Initio Molecular Dynamics Simulations of Extended Systems:  Application to Transition Metal Catalysis. *J Phys Chem B* 1997; **101**: 7877–80.

19. Jarzynski C. Nonequilibrium Equality for Free Energy Differences. *Phys Rev Lett* 1997; **78**: 2690–3.

20. Sprik M, Ciccotti G. Free energy from constrained molecular dynamics. *J Chem Phys* 1998; **109**: 7737–44.

21. Oberhofer H, Dellago C, Geissler P L. Biased Sampling of Nonequilibrium Trajectories:  Can Fast Switching Simulations Outperform Conventional Free Energy Calculation Methods? *J Phys Chem B* 2005; **109**: 6902–15.

22. Ryckaert J -P, Ciccotti G, Berendsen H J C. Numerical integration of the cartesian equations of motion of a system with constraints: molecular dynamics of n-alkanes. *J Comput Phys* 1977; **23**: 327–41.
